# Syntheses and medicinal chemistry of spiro heterocyclic steroids

**DOI:** 10.3762/bjoc.20.152

**Published:** 2024-07-24

**Authors:** Laura L Romero-Hernández, Ana Isabel Ahuja-Casarín, Penélope Merino-Montiel, Sara Montiel-Smith, José Luis Vega-Báez, Jesús Sandoval-Ramírez

**Affiliations:** 1 Facultad de Ciencias Químicas, Benemérita Universidad Autónoma de Puebla, Ciudad Universitaria, 72570, Puebla, Pue., México

**Keywords:** biological activity, drug development, heterocycle, spiro steroid, synthetic transformations

## Abstract

There is compelling evidence that incorporating a heterocyclic moiety into a steroid can alter its pharmacological and pharmacokinetic properties, driving intense interest in the synthesis of such hybrids among research groups. In this review, we present an overview of recent synthetic methodologies, spanning the period from 2000 to 2023, for the preparation of spiro heterocyclic steroids. The compounds surveyed encompass four-, five-, six-, and seven-membered heterocycles appended to various positions of steroidal backbones, with spirocycles containing oxygen, nitrogen, and sulfur atoms being predominant. The outlined synthetic procedures emphasize the pivotal steps for constructing the heterocycles, often accompanied by a detailed account of the overall synthesis pathway. The review encompasses innovative compounds, including bis-steroids linked by a spiro heterocycle and steroids conjugated to heterocyclic moieties containing three or more (hetero)cycles. Moreover, many compounds are accompanied by data on their biological activities, such as antiproliferative, antimalarial, antimicrobial, antifungal, steroid antagonist, and enzyme inhibition, among others, aimed at furnishing pertinent insights for the future design of more potent and selective drugs.

## Introduction

**Figure 1:**
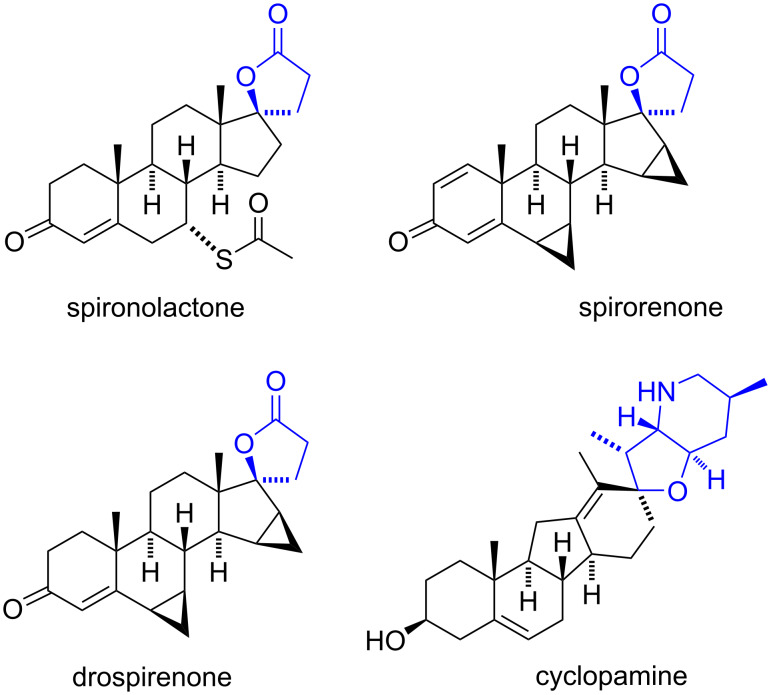
Steroidal spiro heterocycles with remarkable pharmacological activity.

Small-ring heterocycles constitute valuable scaffolds in medicinal chemistry for generating biologically active derivatives. Remarkably, data indicate that at least 85% of all bioactive compounds incorporate a heterocyclic moiety into their structure [1–2]. Similarly, steroids are important structures due to their involvement in numerous therapeutic targets across various organisms. The significance of generating heterocyclic spirosteroids was underscored by the synthesis of spironolactone [3] (Figure 1), which plays a pivotal role in edema treatment. Aldosterone, implicated in water and salt retention in conditions such as congestive heart failure, hypertension, nephrosis, liver cirrhosis, and toxemia of pregnancy, prompted the development of spironolactone as a competitive antagonist of the aldosterone receptor. After this breakthrough, numerous compounds with enhanced anti-aldosterone activity, such as the androstane derivatives spirorenone and drospirenone [4–5] (Figure 1), have been synthesized. The latter has also a progestogen activity, so it is employed in combination with ethinylestradiol in oral contraceptives [6].

Various steroidal spiro heterocycles serve as effective drugs for treating diverse pathologies. For example, cyclopamine (Figure 1) inhibits Hedgehog (HH) signaling by interacting with the 7-transmembrane Smoothened protein (Smo protein) [7]. The HH pathway, implicated in the development and progression of various human cancers (including colorectal, brain, gastrointestinal, lung, and breast cancers), drives tumorigenesis [8]. Consequently, HH pathway inhibitors have demonstrated significant anticancer activity.

Synthesizing spiro heterocyclic steroids entails numerous methodologies. Although the inherent stereochemistry of steroids sometimes facilitates the production of a single diastereomer without the need for chiral auxiliaries, nevertheless, their synthesis remains challenging due to the creation of a new chiral centre. Advances in understanding ligand–receptor interactions have facilitated the determination of optimal molecular conformations to enhance binding affinity, which can be achieved, in part, by introducing a spiro annelated ring to impart rigidity to the molecule.

From a medicinal perspective, the incorporation of a spiro fragment significantly impacts the properties of the drug candidate. Introducing a spirocyclic moiety often leads to increased potency and selectivity, as observed in comparisons between spirocycles and their non-spiro counterparts. Furthermore, several examples illustrate that a spirocycle can alter the stability, physicochemical, and pharmacokinetic properties of the structure [9].

In this review, we summarize recent synthetic procedures reported between 2000 and 2023 for synthesizing steroidal spiro heterocycles. The review categorizes the procedures based on the size and type of targeted cycle, ranging from four- to seven-membered rings, and encompasses heterocycles containing oxygen, nitrogen, sulfur, and/or phosphorus atoms. In instances where the stereochemistry of the new spiro centre remains undetermined, it is omitted. Additionally, the review excludes the steroidal family of spirostans and three-membered spiro heterocyclic steroids, as oxirane derivatives are typically prepared to expand into four- or more membered rings rather than being the final target.

The described methodologies are presented to facilitate the identification of the atoms or functional groups of the steroid involved in heterocycle formation. Many descriptions also include the parent steroid alongside the total steps required to synthesize the target compound. Furthermore, numerous compounds feature data regarding their biological activity, underscoring the remarkable potential of these compounds against various diseases.

## Review

### Spiro steroids with four-membered heterocycles

#### Spirooxetane steroids

**Scheme 1:**
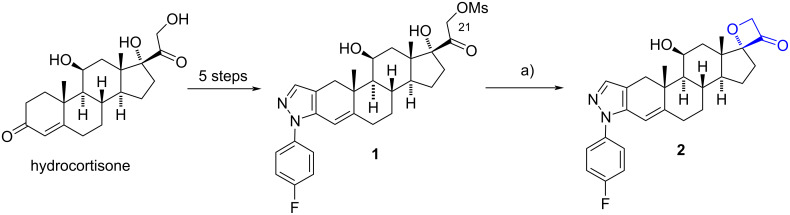
Synthesis of the spirooxetanone **2**. a) *t*-BuOK, THF, rt, 16%.

In 2003 Wüst et al. reported the synthesis of the oxetan-3-one derivative **2** in an overall yield of 4.2% from hydrocortisone, in six steps. A key intermediate for oxetan-3-one **2** was mesylate **1**, whose basic treatment promoted the substitution of the mesylate group at C-21 by the 17α-hydroxy group and the oxetane ring formation in a 16% yield (Scheme 1) [10].

**Scheme 2:**
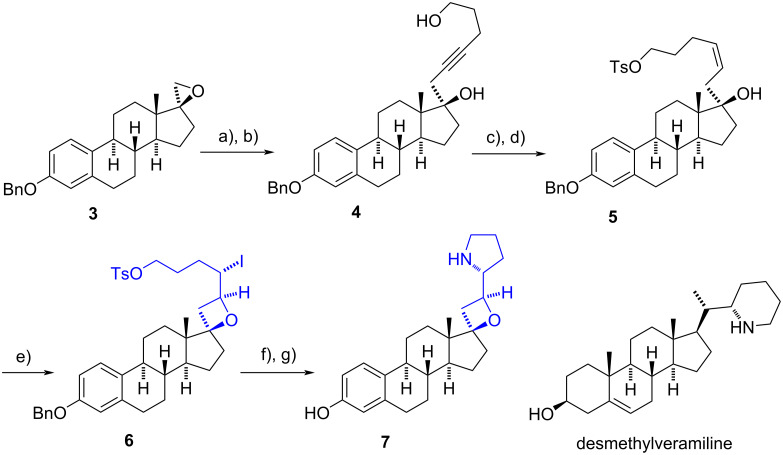
Synthesis of the 17-spirooxetane derivative **7**. a) HC≡C(CH_2_)_2_CH_2_OTBDPS, *n*-BuLi, THF, BF_3_·Et_2_O, −78 °C, 61%; b) TBAF, THF, rt, 95%; c) quinoline, H_2_/Pd/BaSO_4_, EtOAc, rt, 99%; d) DMAP, TsCl, CH_2_Cl_2_, rt, 88%; e) I_2_, NaHCO_3_, MeCN, 0 °C, 64%; f) NH_3_, THF, rt, 77%; g) H_2_, Pd/C, MeOH, rt, 71%.

Winkler et al. developed the synthesis of alkylated derivatives of 17-spirooxetanosteroids from non-alkylated frameworks (Scheme 2) [11]. In 2011, during the synthesis of cyclopamine analogues, the research group constructed a spirooxetane ring on an estrane backbone. The reaction of the spirooxirane of estrane derivative **3** with the anion of TBDPS-protected pentyn-5-ol introduced the pentynyl group, which upon removal of the protecting group yielded the corresponding hydroxyalkynyl derivative **4**. Subsequent Lindlar reduction resulted in the (*Z)*-alkene and a chemoselective tosylation of the primary alcohol led to the formation of tosylate **5**. This intermediate underwent a stereospecific 4-exo cyclization upon exposure to iodine. The oxetane compound **6** was further derivatized to incorporate a pyrrolidine moiety in presence of ammonia. Upon debenzylation at C-3, the target compound **7** was obtained in an 18% overall yield from **3**.

The pyrrolidino-oxetane derivative exhibited potent inhibition of Hedgehog signaling, comparable to cyclopamine. Comparing the biological results of the compound with the activity of desmethylveramiline (a des-E analogue of cyclopamine) has suggested that the rigidity of the oxetane ring is crucial for the potency of compound **7** [12].

#### Spiro-β-lactone steroids

**Scheme 3:**
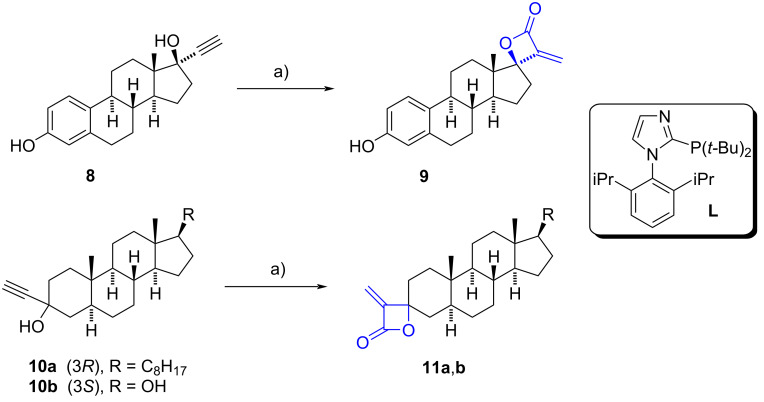
Pd-catalyzed carbonylation of steroidal alkynols to produce α-methylene-β-lactones at C-3 and C-17 positions. a) CO (40 atm), Pd(CH_3_CN)_2_Cl_2_, **L**, MTBE, 100 °C, dr > 20:1, **9** (86%), **11a** (41%), **11b** (52%).

Beller et al. reported a selective method for preparing spiro α-methylene-β-lactones from different steroidal propargylic alcohols [13]. The procedure involves a one-pot Pd-catalyzed cyclocarbonylation of alkynols using 5 mol % of Pd(CH_3_CN)_2_Cl_2_ as a catalyst precursor and 30 mol % of 2-(dibutyl)phosphine-1-(2,6-diisopropylphenyl)-1*H*-imidazole as phosphine-based ligand (**L**). This methodology was applied to the alkynol moiety of ethinylestradiol (**8**) (86% yield), and alkynols derived from ethisterone, levonorgestrel, lynestrenol, and epiandrosterone (epi-ADT), obtaining excellent yields (85–93%) and high diastereoselectivity (dr > 20:1) in all cases. α-Methylene spirolactones at C-3 **11a** and **11b** derived from dihydrocholesterol and stanolone were also obtained in 41% and 52% yields, respectively (dr > 20:1) (Scheme 3).

**Scheme 4:**
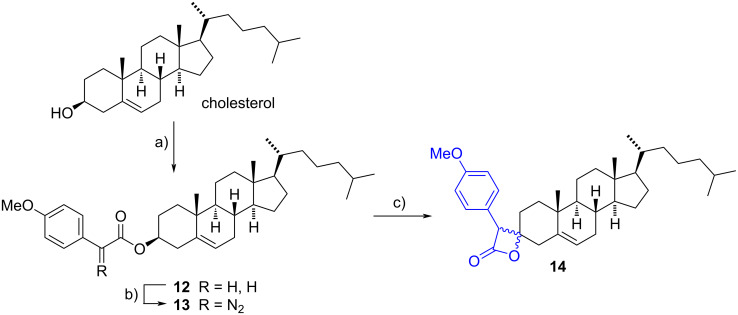
Catalyst-free protocol to obtain functionalized spiro-lactones by an intramolecular C–H insertion. a) 2-(4-Methoxyphenyl)acetic acid, DCC, DMAP, CH_2_Cl_2_, rt, 60%; b) *p*-ABSA, DBU, CH_3_CN, rt, 22%, c) 440 nm blue LEDs irradiation, CH_2_Cl_2_, 50 °C, 80%, 1:1 dr.

More recently, a functionalized spiro-β-lactone was obtained via a metal-free procedure involving photoinduced carbene C–H insertion in a diazo precursor. To achieve this, cholesterol was first esterificated using 2-(4-methoxyphenyl)acetic acid and DCC. The resulting arylacetic ester **12** was then reacted with 4-acetamidobenzenesulfonyl azide (*p*-ABSA) and DBU in anhydrous acetonitrile to yield the diazo compound **13** in 22% yield. The carbene C–H insertion to produce the spiro-β-lactone was accomplished by simply exposing the diazo derivative to 440 nm blue LEDs (Kessil lamp) at 50 °C, that favored the formation of a singlet carbene that reacted selectively by insertion into the C(3)–H bond. Spiro-lactones **14** were obtained in 80% yield as a 1:1 mixture of diastereomers (Scheme 4). The same procedure was also applied to estrone to insert the spiro moiety at C-17 in 70% yield [14].

#### Spiro-β-lactam steroids

**Scheme 5:**
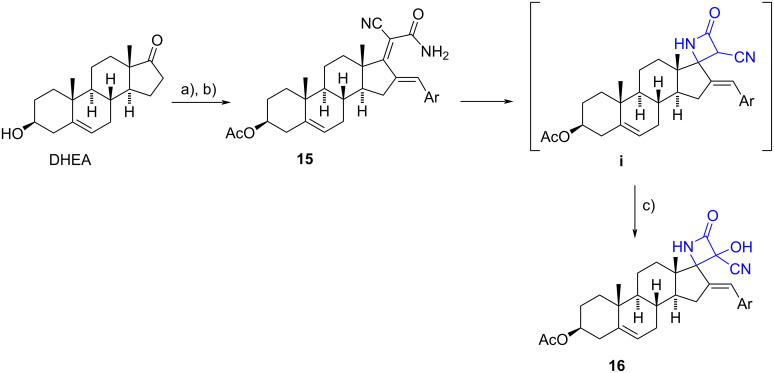
One-pot procedure from dienamides to spiro-β-lactams. a) 1. Ac_2_O, DMAP, Et_3_N, CH_2_Cl_2_, 2. malononitrile, NH_4_OAc, EtOH, reflux, 95%; b) aldehydes, NaOAc, EtOH, reflux, 55–90%; c) NaH, THF, 0 °C, under air atmosphere, 23–68%.

A novel one-pot procedure to build spiro-β-lactams from enamides was described by Liu et al. The research group had previously reported the formation of an α,α-dicyanoalkene from dehydroepiandrosterone (DHEA) and its efficient transformation to dienamides **15** in a single step via a cascade reaction [15–16]. The construction of the β-lactam ring involved a cascade 4-endo *N*-cyclization/aerobic oxidation sequence in presence of sodium hydride, yielding moderate yields (ranging from 23% to 68%). The cyclization initially formed the non-isolated intermediate **i**, which was oxidized by molecular oxygen from air, introducing the hydroxy group at the α-position of the cyano group. The protocol utilised mild conditions and short reaction times. Notably, 6-endo *N*-cyclization to form 2-piperidinones was not observed (Scheme 5).

Using this protocol, a series of spiro-β-lactams were synthesized, with only the structure of the aryl moiety being varied. It is noteworthy to consider that the formation of two vicinal quaternary chiral centres is claimed (C-17 and C-3’). However, no indication was provided regarding their stereochemistry, yet only one stereoisomer was isolated in a pure form.

### Spiro steroids with five-membered heterocycles

#### 17-Spirolactone steroids

**Scheme 6:**
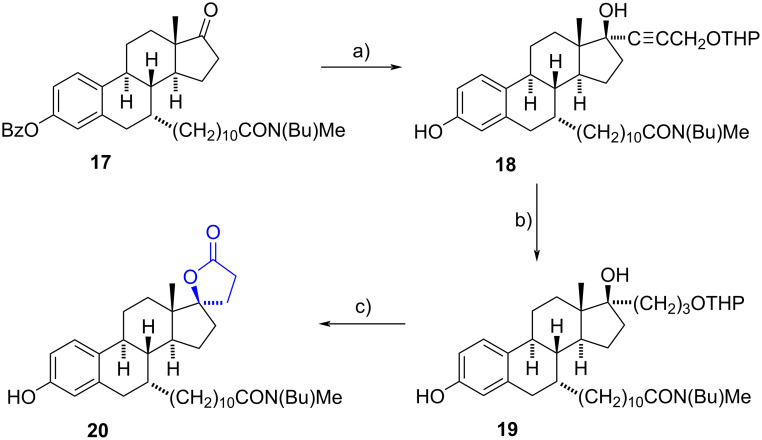
Spiro**-**γ-lactone **20** afforded from 7α-alkanamidoestrone derivative **17**. a) HC≡CCH_2_OTHP, *n*-BuLi, THF, –78 °C, 75%; b) H_2_, Pd/C, Pd/CaCO_3_, EtOAc, rt, 92%; c) 1. Amberlyst-15^®^ resin, MeOH, rt, 2. Jones reagent, acetone, 0 °C, 48%.

In 2000**,** Poirier’s group reported an efficient synthesis of a spiro-γ-lactone motif on an estradiol backbone [17]. Beginning with the 7α-alkanamidoestrone derivative **17**, a nucleophilic addition by the anion of the THP propargyl ether occurred stereoselectively and provided the alkyne **18** in a 75% yield. Afterwards, the catalytic hydrogenation of the alkyne with a 1:1 mixture of 10% Pd/C and 5% Pd/CaCO_3_ yielded reduced compound **19**. Cleavage of the THP group with Amberlist-15^®^ resin and a final Jones oxidation of the primary alcohol to the carboxylic acid, led to cyclization with the 17β-OH group affording the lactone **20** in a moderate overall yield (Scheme 6). Biological assays demonstrated that the γ-spirolactone **20** inhibited the activity of 17β-hydroxysteroid dehydrogenase type 2 (17β-HSD2) with an IC_50_ value of 0.35 µM.

In 2004, the same research group reported the synthesis of similar spiro-γ-lactones from 17α- and 17β-allylestradiols (not substituted at C-7). The compounds were hydrogenated, oxidized, and lactonized in a similar fashion, utilizing either the Jones reagent or manganese dioxide as the oxidizing agent [18].

**Scheme 7:**
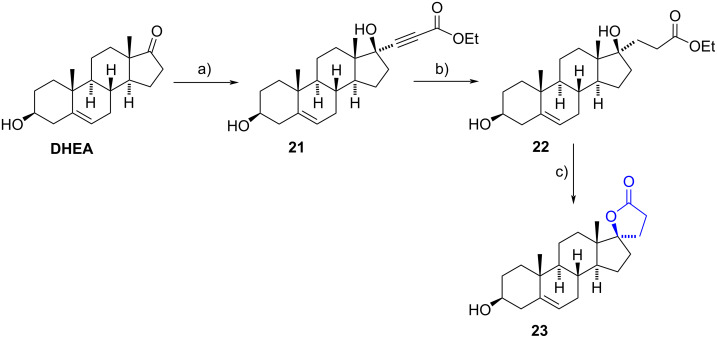
Synthesis of the 17-spiro-γ-lactone **23**, a key intermediate to obtain spironolactone. a) Ethyl propiolate, LiHMDS, THF, −65 °C to −40 °C, 94%; b) H_2_, 5% Pd/C, THF, rt, 98%; c) TsOH, THF/H_2_O, rt, 96%.

Santhamma et al. developed another route for steroidal spiro-γ-lactones at C-17, from estran-17-one and androstane-17-one derivatives [19]. The steroidal 17-ketones were first alkylated in the presence of the lithium derivative of ethyl propiolate. After stereoselective formation of the corresponding adduct, the triple bond was chemoselectively reduced under catalytic hydrogenation using 5% palladium on charcoal. As a final step, a *p*-toluenesulfonic acid-mediated hydrolysis led to the cyclization reaction yielding the steroidal 17-spiro derivatives. The procedure was applied to synthesize drospirenone (Figure 1) and extended to build the spirolactone **23** from DHEA, a key intermediate toward the popular diuretic spironolactone (Scheme 7).

#### 17-Spirofuran steroids

**Scheme 8:**
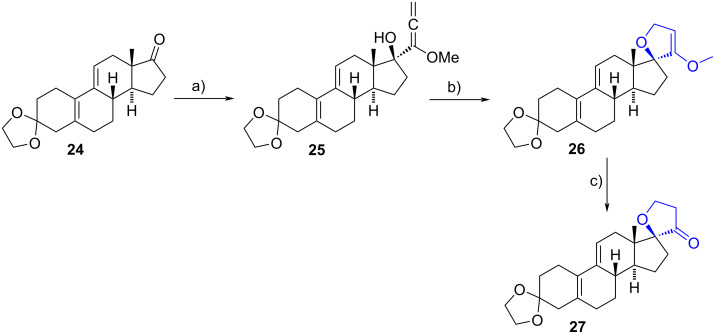
Synthetic pathway to obtain 17-spirodihydrofuran-3(2*H*)-ones from 17-oxosteroids. a) 1-Methoxypropa-1,2-diene, *n*-BuLi, THF, −78 °C; b) dicyclohexyl-18-crown-6, *t*-BuOK, *t*-BuOH; c) HCl, acetone, 43% from **24**.

Intramolecular cyclization of alcohols with allenes is a common method for obtaining 17-spirodihydro-(2*H*)-furan-3-ones. In 2006, Jiang et al. utilized a known reaction sequence to construct the 17-spiro heterocycle **27** from the commercially available ethylene deltenone **24** [20–21]. In this process, the carbonyl group at C-17 was stereoselectively attacked by α-lithio-α-methoxyallene at −78 °C to produce allene **25**. A further cyclization reaction was induced by potassium *tert*-butoxide in the presence of catalytic dicyclohexyl-18-crown-6. The final 17-spirodihydro-(2*H*)-furan-3-one **27** was obtained in a 43% overall yield after acid hydrolysis (Scheme 8).

**Scheme 9:**
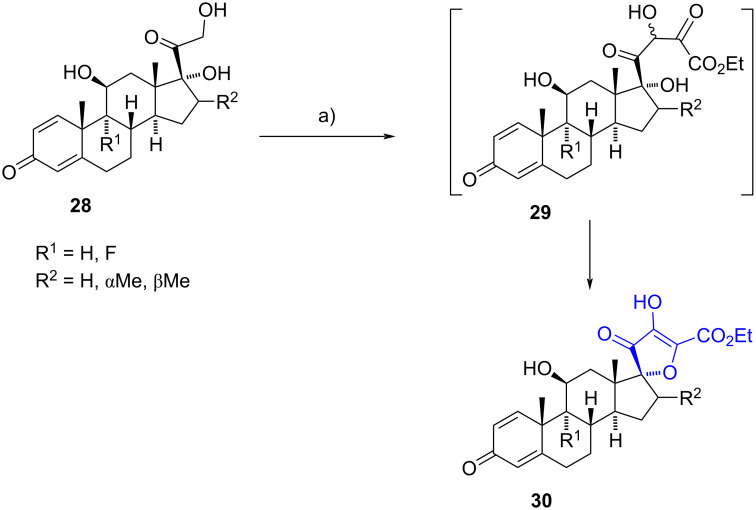
One-pot procedure to obtain 17-spiro-2*H*-furan-3-one compounds. a) NaH, diethyl oxalate, benzene, rt, 29–50%.

Unsaturated spiro-2*H*-furan-3-ones have been synthesized using various procedures. In 2000, Lee et al. employed an intramolecular condensation reaction involving an α-ketoester derived from prednisolone precursors to produce the target spiro-2*H*-furan-3-one framework at position C-17 [22]. The one-pot procedure involved the condensation of diethyl oxalate with the α-hydroxy ketone moiety of derivatives **28**, immediately followed by a cyclization between the 17α-hydroxy group and the carbonyl group of the α-ketoester in **29**. Spiro compounds **30** were obtained in moderate yields (ranging from 29% to 50%, Scheme 9) and were tested as anti-inflammatory drugs in RAW 264.7 cells and as inhibitors of rat ear edema. Unfortunately, the tested compounds exhibited weak activity in all assays.

**Scheme 10:**
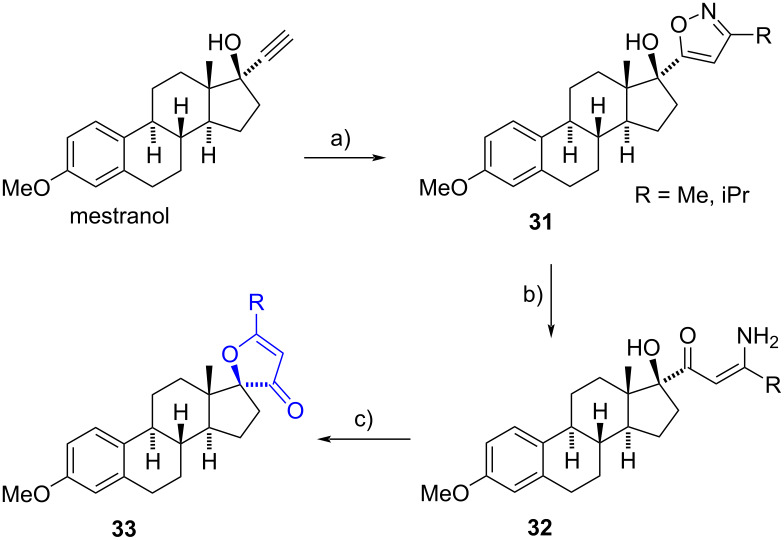
Synthesis of 17-spiro-2*H*-furan-3-one derivatives. a) RCH=NOH, *N*-chlorosuccinimide/CHCl_3_, 99%; b) H_2_, Raney-Ni, H_3_BO_4_, EtOH, rt; c) HCl, EtOH, rt, 57–64% (from **31**).

Khripach et al. reported a three-step sequence for the synthesis of 2*H*-furan-3-ones from mestranol [23]. Initially, a 1,3-dipolar regioselective cycloaddition occurred between the alkyne group of mestranol and an alkanonitrile oxide generated in situ from the corresponding oxime, yielding isoxazoles **31**. Later, a reductive ring cleavage of the isoxazole ring was conducted via catalytic hydrogenolysis, resulting in the formation of enaminoketones **32**. Finally, enaminoketones were subjected to acid hydrolysis conditions facilitating ring closure and producing the spiro-2*H*-furan-3-ones **33** in moderate yield (Scheme 10). This procedure was also applied to norethisterone with similar yields.

In 2006, Akita et al. used an intramolecular Knoevenagel–Claisen type condensation between a β-ketoester and an acetate residue to synthesize spiro-2*H*-furan-3-ones [24]. For instance, the intermediate orthoester **35** was obtained in 86% yield after a cyclization–carbonylation reaction of mestranol acetate (**34**) catalysed by a palladium(II) complex, in the presence of *p*-benzoquinone under carbon monoxide. The authors propose an initial addition of the acetate C=O group onto the triple bond coordinated to palladium, followed by a MeOH attack onto the resulting oxycarbenium, and a final CO insertion to release the ester. After an acid hydrolysis which produced **36**, a basic treatment induced the condensation reaction to yield the heterocycle **37** in 99% yield.

**Scheme 11:**
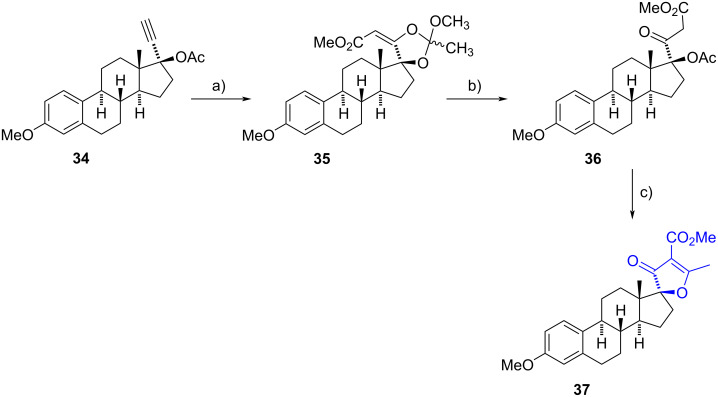
Intramolecular condensation of a γ-acetoxy-β-ketoester to synthesize spirofuranone **37**. a) (CH_3_CN)_2_PdCl_2_, CO, *p*-benzoquinone, MeOH, 0 °C, 86%; b) 10% HCl, MeOH, rt, 98%; c) NaHCO_3_, MeOH, rt, 99%.

This reaction sequence was also applied to the acetates of ethisterone and ethynylestradiol, resulting in similar yields in all cases. All spirofuranones exhibited inhibitory activity on CYP3A, a well-known effect of steroids with an acetylene moiety at C-17. Additionally, compound **37** demonstrated vasorelaxant and bradycardiac effects when tested in precontracted aortic rings (Scheme 11).

**Scheme 12:**
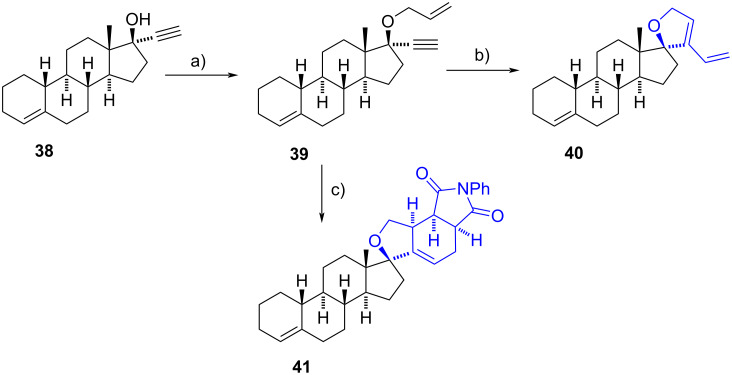
Synthesis of spiro 2,5-dihydrofuran derivatives. a) Allyl bromide, DMF, NaH, 0 °C to rt, 93%; b) G-II, toluene, MW 120 °C, 76%; c) 1. G-II, toluene, MW 120 °C, 2. *N*-phenylmaleimide, MW 150 °C, 84%.

More recently, Nay’s group reported a novel and straightforward method for synthesizing spiro 2,5-dihydrofuran derivatives starting from 17-ethynyl-17-hydroxysteroids such as lynestrenol (**38**) (Scheme 12) [25]. The 17-hydroxy group of steroids underwent allylation using allyl bromide and sodium hydride. After formation of the alkenyl ether **39**, a ring-closing enyne metathesis (RCEYM) was initiated using the Grubbs second-generation catalyst (G-II) and high temperature to obtain the spiro 2,5-dihydrofuran derivative **40** in 76% yield. Additionally, when a dienophile such as *N*-phenylmaleimide was directly added to the same pot and microwaved, a Diels–Alder reaction occurred, yielding the spiro product **41**. The reaction conditions were also applied to 17-ethynyl-17-hydroxysteroids derived from mestranol and desogestrel obtaining similar results. The one-pot RCEYM/Diels–Alder reaction was only applied to mestranol and lynestrenol derivatives, affording products in yields ranging from 10% to 91%, depending on the structure of the dienophile.

#### Spiropyrrolidine steroids

**Scheme 13:**
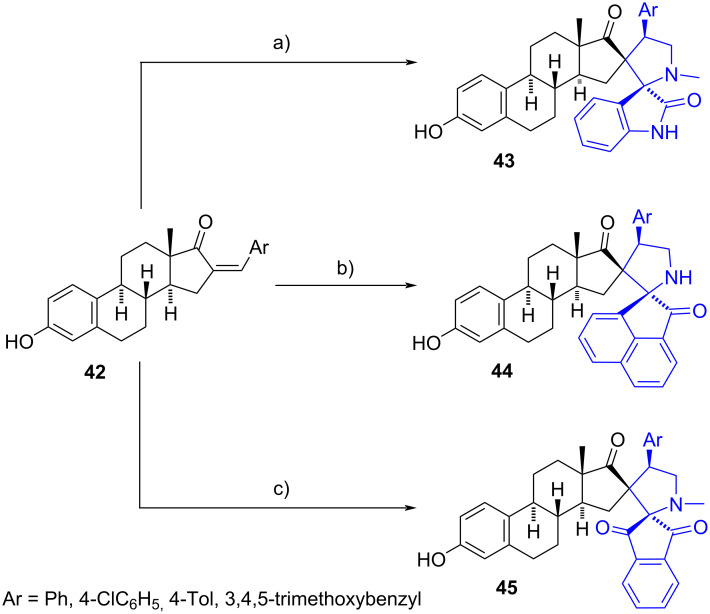
First reported synthesis of C-16 dispiropyrrolidine derivatives. a) Sarcosine, isatin, MeOH, reflux, 67–70%; b) sarcosine, acenaphthenequinone, MeOH, reflux, 66–72%; c) sarcosine, ninhydrin, MeOH, reflux, 68–70%.

Numerous dispiropyrrolidine derivatives have been reported on various steroidal nuclei, particularly at C-16. Raghunathan and Babu were the first to describe the synthesis of these compounds via an intramolecular [3 + 2] cycloaddition reaction between (*Z*)-steroidal arylidene derivatives **42** and azomethine ylides (Scheme 13) [26]. The latter were generated in situ from sarcosine (MeNHCH_2_CO_2_H) and a mono- or dicarbonyl compound such as isatin, acenaphthenequinone, and ninhydrin.

Estrone-derived dispiropyrrolidines **43**, **44**, and **45** were obtained in a regio- and stereoselective manner (ylide attacks by the α-steroidal face) in yields ranging from 66% to 72%.

**Scheme 14:**
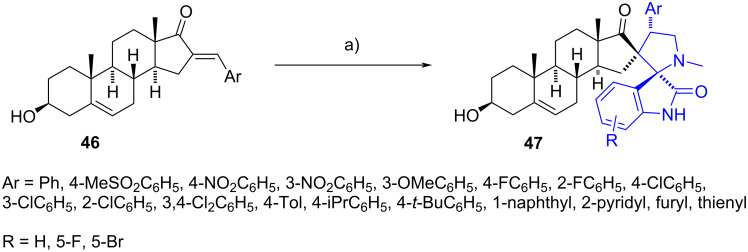
Cycloadducts **47** with antiproliferative activity against human cancer cell lines. a) 1,4-Dioxane–MeOH, isatin, sarcosine, heat, 73–93%.

1,3-Dipolar cyclization of azomethine ylides can also be achieved using (*E*)-steroidal arylidenes at C-16. In 2014, Liu et al. reported the synthesis of cycloadducts **47** from reaction of (*E*)-16-arylidene derivatives **46** and azomethine ylides generated in situ from substituted isatins and sarcosine [27] (Scheme 14). The resulting spiro compounds proved to be potent antiproliferative agents against human cancer cell lines with IC_50_ values ranging from 0.7 to 43 µM, some of them exhibiting significantly more potent activity than the reference compound 5-fluorouracil (5-FU). More recently, similar compounds were evaluated for their cytotoxicity using a brine shrimp bioassay, with LC_50_ values ranging from 6.19 to 27.2 µg/mL. Since the cytotoxicity of the parent 16-arylidene steroids was LC_50_ > 100 µg/mL, it was concluded that the presence of the pyrrolidine moiety was essential for the activity [28]. Additional derivatives have been reported on different steroidal positions [29] or with variations in the methodology, such as a one-pot four-component synthesis and the use of ionic liquids as solvents [30].

**Scheme 15:**
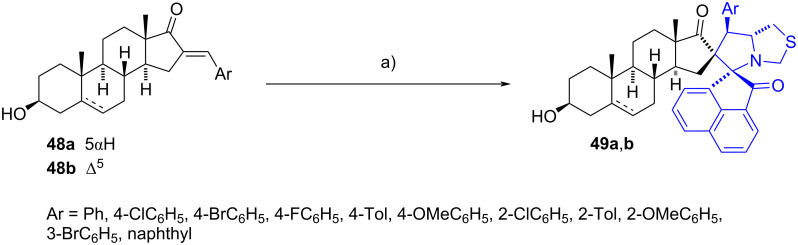
Spiropyrrolidine compounds generated from (*E*)-16-arylidene steroids and different ylides. a) Acenaphthenequinone 1,3-thiazolidine-4-carboxylic acid, MeOH, reflux, 75–91%.

Kanchithalaivan et al. [31] reported a library of 16-spiro pyrrolo[1,2-*c*][1,3]thiazoles of *trans*-androsterone and DHEA (**49a** and **49b**, respectively). The syntheses were achieved through the 1,3-dipolar cycloaddition of a set of azomethine ylides and the 16-arylidene steroids **48a**,**b**. Ylides were generated in situ by the nitrogen attack of the thiazolidine ring from 1,3-thiazolidine-4-carboxylic acid to a carbonyl group of acenaphthenequinone. The resulting reaction produced an iminium group, which promoted the decarboxylation of the carboxylic residue when heated at reflux of methanol. After the regioselective cycloadditions, spiro compounds **49a**,**b** were obtained in yields ranging from 75% to 91% (Scheme 15). Related derivatives have similarly been achieved in good to excellent yields [32–33].

**Scheme 16:**
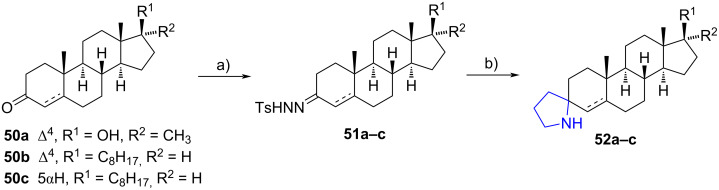
3-Spiropyrrolidines **52a**–**c** obtained from ketones **50a**–**c**. a) *p*-Toluenesulfonyl hydrazide, MeOH, rt; b) (3-azidopropyl)boronic acid, K_2_CO_3_ or Cs_2_CO_3_, 1,4-dioxane or chlorobenzene, MW 150 °C, dr = 1:1, **52a** (89% from **50a**), **52b** (85% from **50b**), **52c** (80% from **50c**).

Recently, López et al. reported the synthesis of spiropyrrolidines at the ketone group of 17α-methyltestosterone (**50a**), cholest-4-en-3-one (**50b**), 5α-cholestan-3-one (**50c**), and 3-MOMO-estrone [34]. To construct the spiro heterocycle, an addition of *p*-toluenesulfonyl hydrazide to the carbonyl group of compounds **50a**–**c** was carried out, resulting in the corresponding *N*-tosylhydrazones **51a**–**c**. Afterwards, the compounds were subjected to microwave irradiation in the presence of (3-azidopropyl)boronic acid and potassium or cesium carbonate, yielding 3-spiropyrrolidines **52a**–**c** in high overall yields as 1:1 mixture of diastereomers (Scheme 16). Remarkably, the hydroxy group at **50a** remained unprotected. Additionally, the isomers of **52b**,**c** were cleanly separated on neutral alumina. In the case of the estrone derivative, the spirocycle was built at C-17 in only 30% yield, not being the main product, but a rearranged deoxygenated starting material (35%).

#### 16-Spiropyrazoline steroids

**Scheme 17:**
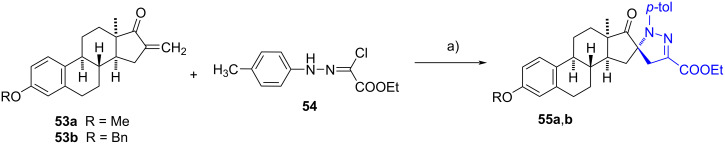
16-Spiropyrazolines from 16-methylene-13α-estrone derivatives. a) AgOAc, toluene, rt, 78–81%.

In 2009 Mernyák et al. described the synthesis of 16-spiropyrazolines from 16-methylene-13α-estrone derivatives via a 1,3-dipolar cycloaddition reaction between the methylene of α,β-unsaturated ketones **53a**,**b** and the ylide produced from the tolylhydrazonoyl chloride derivative **54** [35]. The reaction conducted in the presence of silver acetate at room temperature, achieved yields of 78–81% of the 16-spiropyrazolines **55a**,**b** (Scheme 17).

#### 6-Spiroimidazoline steroids

**Scheme 18:**
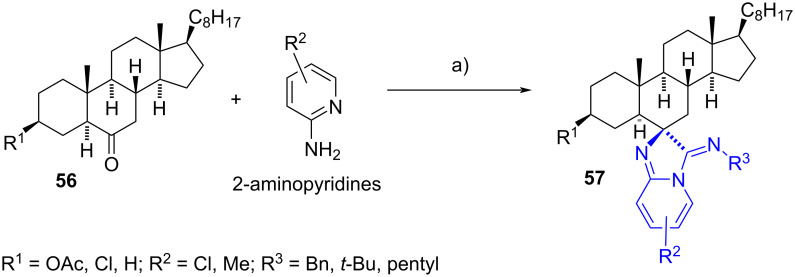
6-Spiroimidazolines **57** synthesized by a one-pot multicomponent reaction. a) R^3^-NC, T3P^®^, DMSO, 70 °C, 85–90%.

In 2015, Dar’s group reported a small library of spiroimidazo[1,2-*a*]pyridines obtained through a one-pot multicomponent reaction (MCR) involving the reaction of substituted 2-aminopyridines, isocyanides, and the cholestanone derivatives **56** [36]. The reactions were conducted in dimethyl sulfoxide at 70 °C, and catalysed by propylphosphonic anhydride (T3P^®^), providing high yields in all cases. Chromatographic purification was not required post-reaction. Some spiro products exhibited high binding affinity towards DNA, while others showed good cytotoxicity against different cancer cells (A545, MCF-7, HeLa, HL-60, SW480, HepG2, HT-29, and A549) with IC_50_ values within the micromolar range (2.18–18.54 µM) (Scheme 18).

#### 17-Spiro-1,3-oxazoline steroids

Jin et al. [37] described a series of spiro-1,3-oxazolines synthesized from the commercially available estrane derivative ethylene deltenone **24**. This conjugated diene was regioselectively epoxidized at the 5(10) double bond to furnish compound **58**. Amidoacetylenes **59** were synthesized after eight synthetic steps, and subsequently subjected to a copper(I)-catalysed cyclization to produce 17-spiro-1,3-oxazolines **60** in moderate to excellent yields (38–97%). The cyclization of acetamido and propionamido residues gave much better results than the use of formamido and trifluoroacetamido groups in almost all cases.

**Scheme 19:**
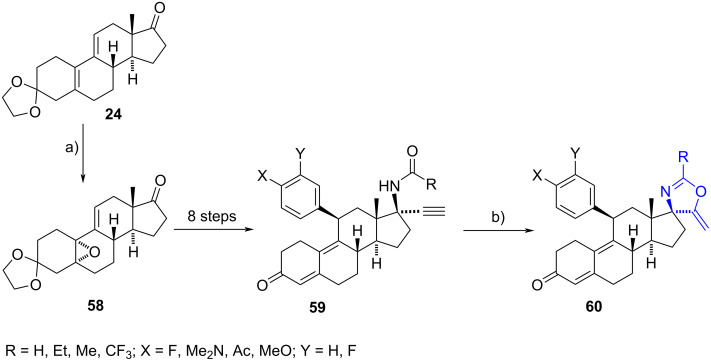
Synthesis of spiro-1,3-oxazolines **60**, tested as progesterone receptor antagonist agents. a) CF_3_COCF_3_, Na_2_HPO_4_, H_2_O_2_, CH_2_Cl_2_, 0 °C to rt, 53%; b) 10 mol % CuI, benzene–Et_3_N, 90 °C, 38–97%.

Spirooxazolines **60** were evaluated as progesterone receptor (PR) antagonist agents, comparing their activity to the abortifacient mifepristone, which has undesired glucocorticoid antagonist activity. The compounds exhibited high PR antagonist activity, albeit less potent than the reference drug. However, many oxazolines showed improved selectivity, with greater separation of antiprogestational and antiglucocorticoid activity (Scheme 19) [38].

1,2-Aminoalcohols have been extensively utilized as building blocks for synthesizing spiro-1,3-oxazolidin-2-ones (spirocarbamates). These spiro heterocycles are commonly obtained in good yields through treatment with triphosgene and DIPEA.

**Scheme 20:**
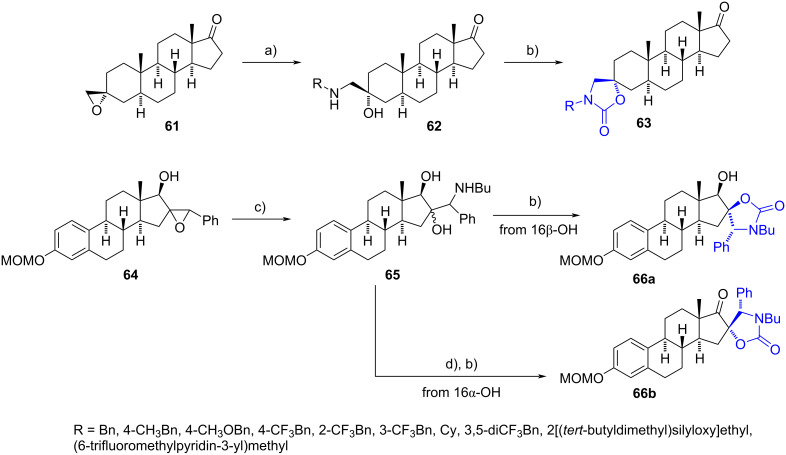
Synthesis of spiro-1,3-oxazolidin-2-ones **63** and **66a**,**b**. a) RNH_2_, EtOH, 70 °C, 70–90%; b) (CCl_3_O)_2_CO, DIPEA, DCM, rt, 63 (14–66%), **66a** (86%), **66b** (80%); c) *n*-BuNH_2_, EtOH, 180 °C MW, 65–99%; d) PS-IBX, TFA, DCM, rt.

In the last decade, Poirier’s group reported a synthetic strategy to produce several spiro derivatives as discovery platforms for new bioactive compounds (Scheme 20) [39–40]. Following this procedure, a series of substituted spirocarbamates were obtained at C-3 and C-16 positions from aminoalcohols **62** and **65**, generated from the opening of the corresponding epoxides **61** and **64**. In the case of derivative **66b**, a prior oxidation of the 17β-hydroxy group of **65**, using the polymeric version of 2-iodoxybenzoic acid (PS-IBX), was conducted to block the formation of the six-membered ring.

Spiro-1,3-oxazolidinones **63** were assessed as inhibitors of 17β-HSD type 3, an enzyme involved in testosterone and dihydrotestosterone synthesis. The structure–activity relationship revealed that compounds bearing hydrophobic fragments were superior inhibitors compared to those with polar groups. Additionally, some derivatives exhibited better activity than the reference compound, with more than 40% inhibition of the enzyme. The research group also described additional spiro products synthesized similarly and their evaluation as inhibitors of the same enzyme [41–42], many of which demonstrated activity comparable to the inhibitor RM-532-105 [43].

**Scheme 21:**
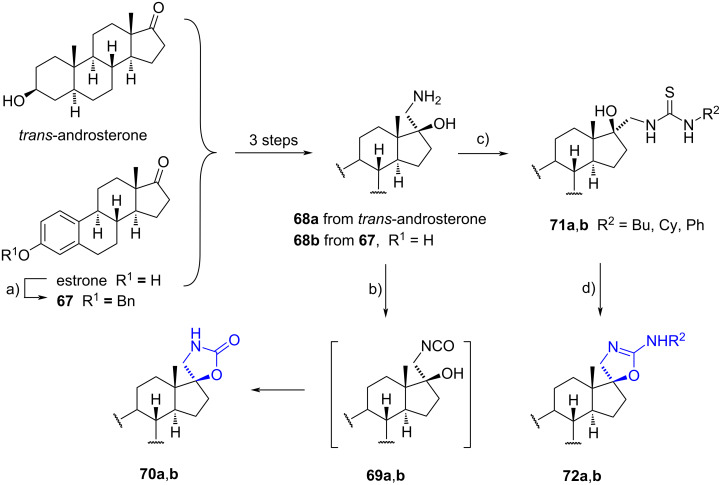
Formation of spiro 1,3-oxazolidin-2-one and spiro 2-substituted amino-4,5-dihydro-1,3-oxazoles from *trans*-androsterone and estrone. a) BnBr, K_2_CO_3_, CH_3_CN, reflux, 99%; b) (CCl_3_O)_2_CO, MeOH–NaHCO_3_ aq, 0 °C, **70a** (36% from *trans*-androsterone), **70b** (44% from **67**); c) R-NCS, Et_3_N, MeOH, rt or 45 °C, **71a** (70–81%), **71b** (66–86%); d) HgO, MeOH or THF, rt, dark, **72a** (93–99%), **72b** (68–96%).

In 2018, Merino-Montiel and Montiel-Smith prepared spiro 1,3-oxazolidin-2-ones (spirocarbamates) and a series of spiro 2-substituted amino-4,5-dihydro-1,3-oxazoles at the C-17 position of *trans*-androsterone and estrone [44]. The synthesis of these spiro compounds was achieved using aminoalcohols **68a**,**b** derived from those steroids. Treatment of aminoalcohols with triphosgene led to the formation of isocyanates **69a**,**b**, which spontaneously cyclized to provide spirocarbamates **70a**,**b** in moderate yields. Alternatively, spiro 2-alkylamino- and 2-arylamino-4,5-dihydro-1,3-oxazoles **72a**,**b** were obtained by treating aminoalcohols **68a**,**b** with isothiocyanates in the presence of triethylamine. The resulting thioureas **71a**,**b** underwent cyclodesulfurization promoted by mercury(II) oxide, yielding spiro 2-substituted amino-4,5-dihydro-1,3-oxazoles **72a**,**b** in moderate to good yields (Scheme 21).

All spiro derivatives exhibited potent antiproliferative activity when tested on six human cancer cell lines (GI_50_ = 0.34–18 µM). Structure–activity relationships revealed that compounds derived from estrone were more potent than those from *trans*-androsterone. Additionally, spiro 2-aminooxazolines were identified as the most active compounds in both series. Notably, when considering the substituent on the amino group, butyl and cyclohexyl derivatives displayed superior results compared to phenyl-substituted compounds.

Frank et al. synthesized a series of 16-spiroisoxazolines through a regio- and a highly stereoselective 1,3-dipolar cycloaddition between the double bond of the α,β-unsaturated steroidal ketone **73** and various arylnitrile oxides [45]. The reaction primarily resulted in the formation of isomers **74**, in which the positive carbocation of the ylide was attacked by the double bond of methylene (C-16^1^), followed by the addition of the negative oxygen atom of the dipole. This cycloaddition occurred highly selectively on the α-side of the double bond. Minor quantities of diastereomers **75**, derived from β-side attack, were also observed.

**Scheme 22:**
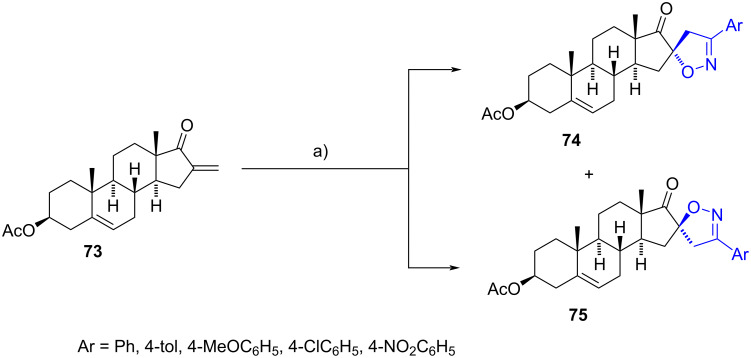
Synthesis of diastereomeric spiroisoxazolines **74** and **75**. a) Ar-C(Cl)=N-OH, DIPEA, toluene, rt, **74** (71–89%), **75** (6–10%).

Subsequently, spiroisoxazolines **74** and **75** were assessed for their antiproliferative activity in three malignant human cell lines (HeLa, MCF7, and A431). The data revealed that the aryl compounds **74** exhibited greater potency compared to the stereoisomers **75**, with enhanced activity observed upon substitution of the aromatic ring. Interestingly, hydrolysis of the acetate group on C-3 did not significantly affect the antiproliferative effect, indicating that the activity is primarily influenced by the stereochemistry and substituents of the new heterocycle rather than functionalization on C-3 (Scheme 22).

#### Spirothiazolidine steroids

**Scheme 23:**
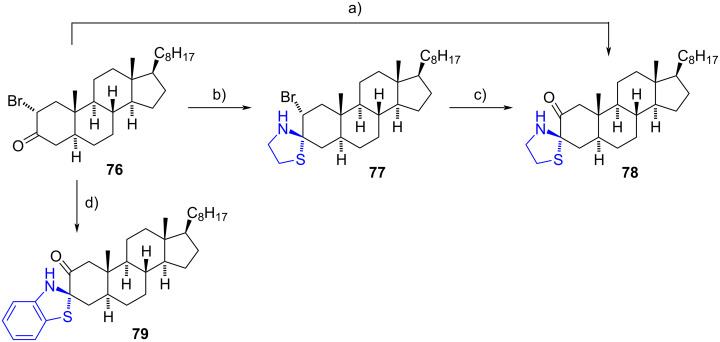
Spiro 1,3-thiazolidine derivatives **77**–**79** obtained from 2α-bromo-5α-cholestan-3-one **76**. a) 2-aminoethanethiol, benzene, 168 h, rt, 66%; b) 2-aminoethanethiol, pyridine, rt, 1.5 h, 22%; c) 2-aminoethanethiol, pyridine, rt, 100 h, 48%; d) 2-aminobenzenethiol, pyridine, 100 h, rt, 61%.

Horiuchi et al. introduced a novel synthetic approach for the preparation of steroidal spiro 1,3-thiazolidines from 2α-bromo-3-oxo steroids, exemplified by compound **76** [46]. Initially, treatment of 2α-bromo-5α-cholestan-3-one (**76**) with 2-aminoethanethiol in benzene led to the formation of the spiro 1,3-thiazolidine 3-oxosteroid derivative **78** in a stereoselective manner, with a notable 66% yield. The authors proposed that after the formation of the spiro 1,3-thiazolidine **77**, an autooxidation process occurs via a 1,4-thiazine intermediate. Alternatively, compound **78** could be obtained from the isolated intermediate bromo-compound **77**. When 2-aminobenzenethiol was employed, derivative **79** was obtained with a satisfactory yield of 61% (Scheme 23). The reaction conditions were effective for various 2α-bromo-3-oxo-, 2β-bromo-3-oxo-, and 4β-bromo-3-oxo-steroids.

**Scheme 24:**
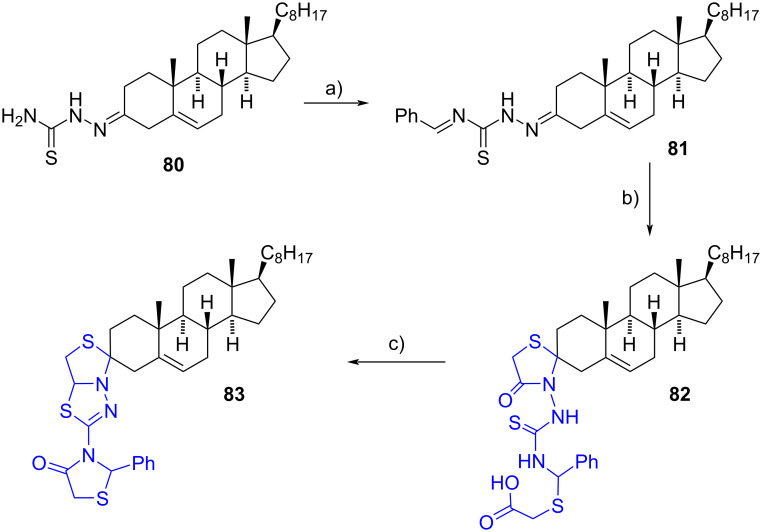
Method for the preparation of derivative **83**. a) Benzaldehyde, MeOH, reflux, 77%; b) thioglycolic acid, 1,4-dioxane, reflux, 70%; c) 1. conc. H_2_SO_4_, 2. liq. NH_3_, 60%.

In 2012, Shamsuzzaman et al. presented a synthetic route for the preparation of novel steroidal spiro derivatives incorporating three heterocyclic moieties [47]. Initially, thiosemicarbazone **80** served as the starting material, undergoing a condensation reaction with benzaldehyde to yield the Schiff base 1’-benzylidine-4’-(cholest-5-ene-3-yl)thiosemicarbazone (**81**) in 77% yield. Subsequently, the resulting product was subjected to a reaction with thioglycolic acid in dioxane, affording intermediate **82** in 70% yield. Upon treatment of **82** with concentrated sulfuric acid, cyclodehydration occurred, leading to the formation of 3-spiro[3’’’-(2’’’-phenyl-4’’’-oxo-1’’’,3’’’-thiazolidin-5’’-yl)-1’’,3’’,4’’-thiadiazolo[3’,4’-*b*]thiazolin]cholest-5-ene (**83**) in 60% yield (Scheme 24). Notably, stereochemical information at the newly formed stereocenter C-3 was not provided.

**Scheme 25:**
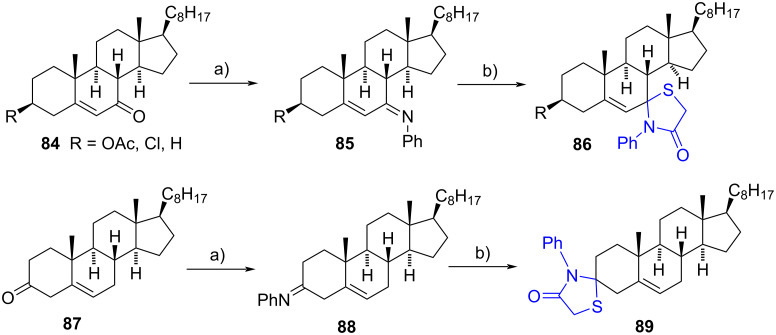
Synthesis of spiro 1,3-thiazolidin-4-one derivatives from steroidal ketones. a) Aniline, EtOH, reflux, b) SHCH_2_COOH, benzene, reflux.

The same research group later reported a method for synthesizing steroidal spiro 1,3-thiazolidin-4-one derivatives in a two-step process starting from α,β-unsaturated ketones [48]. Initially, steroidal iminophenyl compounds **85** were synthesized in yields ranging from 70% to 75% by condensing unsaturated ketones **84** with aniline under refluxing ethanol. These intermediates were then subjected to a reaction with excess mercaptoacetic acid (also known as thioglycolic acid) in refluxing benzene, resulting in the formation of spiro 1,3-thiazolidin-4-one derivatives with good yields in all cases (Scheme 25). The same methodology was applied to prepare spiro 1,3-thiazolidin-4-one **89**, at the C-3 steroidal position from cholest-5-en-3-one (**87**), yielding an overall yield of 55%. The stereochemical information regarding the new chiral carbons was not provided in any instance.

The synthesized compounds were evaluated for their DNA binding properties and screened for cytotoxicity against leukemia cancer cells (Jurkat), demonstrating IC_50_ values in the micromolar range (14.2 to 36.5 µM). Importantly, these derivatives exhibited minimal toxicity toward normal cells (PBMCs). Furthermore, the compounds demonstrated significant acetylcholinesterase inhibitory activity, with IC_50_ values ranging from 0.35 to 0.50 µM compared to the reference inhibitor tacrine (IC_50_ value = 0.29 µM).

**Scheme 26:**
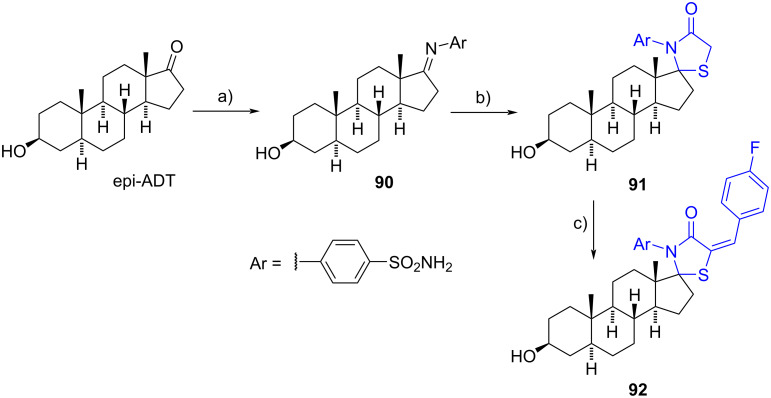
Synthesis of spiro *N*-aryl-1,3-thiazolidin-4-one derivatives **91** and **92**. a) Sulfanilamide, DMF, reflux, 60%; b) thioglycolic acid, 1,4-dioxane, reflux, 65%; c) 4-fluorobenzaldehyde, piperidine, EtOH, reflux, 70%.

In 2016, El-Shahawi et al. presented the synthesis of novel spiro 1,3-thiazolidin-4-one derivatives at the C-3 or C-17 steroidal positions [49]. As an example, epi-androsterone was condensed with sulfanilamide resulting in the formation of substituted imine **90**. Subsequent cycloaddition with thioglycolic acid under refluxing conditions in dioxane furnished the spiro *N*-aryl-1,3-thiazolidin-4-one **91**. This compound underwent further transformation through reaction with *p*-fluorobenzaldehyde in dry ethanol, yielding the corresponding 4-fluoroarylidene derivative **92** in an overall yield of 27%. The described reaction sequence was successfully replicated using testosterone and progesterone to produce a *N*-aryl-1,3-thiazolidin-4-one moiety at the C-3 steroid position, employing the drugs sulfapyridine and sulfadiazine. The corresponding heterocycles were afforded in similar yields. The spiro products were evaluated for their antimicrobial and antifungal properties, demonstrating comparable activity to the antibiotic piperacillin and the antifungal nystatin (Scheme 26). However, no information regarding the stereochemistry at C-17 was provided.

#### Spiro-1,2,4-trithiolane steroids

**Scheme 27:**
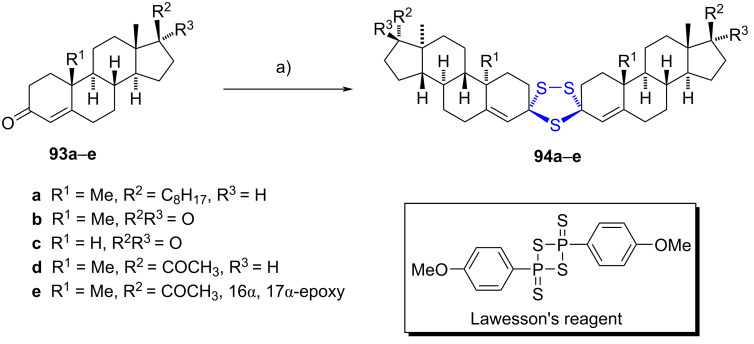
1,2,4-Trithiolane dimers **94a**–**e** selectively obtained from carbonyl derivatives. a) LR, CH_2_Cl_2_, reflux, 4–8 h, 11–79%.

Krstić et al. elucidated the reaction involving various α,β-unsaturated cholestane, androstane, and pregnane carbonyl derivatives **93a**–**e** with Lawesson's reagent (LR), yielding chemoselectively 1,2,4-trithiolane dimers **94a**–**e** [50]. The reaction led to the formation of diverse sulfur products, including (di)thioketones, dimeric sulfides, and (4-methoxyphenyl)phosphonotrithioates contingent on reaction time and solvent used. Refluxing unsaturated ketones **93a**–**e** for 4–8 hours in dichloromethane predominantly produced dimers **94a**–**e** featuring a 1,2,4-trithiolane ring as the primary products, with yields ranging from 11% to 79% (Scheme 27).

Spirodimers **94a**–**e** demonstrated significant bioactivity against *C. albicans* (ATCC 10231) and *S. cerevisiae* (ATCC 9763), displaying selective antifungal activity akin to nystatin, alongside with low cytotoxicity on cancer cell lines (HeLa, MDA-MB-453, and MDA-MB-361). Additionally, they exhibited robust activity against leukemia K562 cells [51].

#### Spiro-1,2,4-triazolidin-3-one steroids

**Scheme 28:**
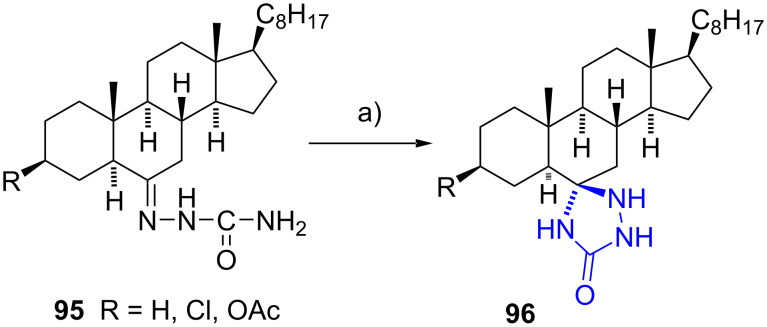
Spiro 1,2,4-triazolidin-3-ones synthesized from semicarbazones. a) H_2_O_2_, CHCl_3_, 0 °C, 82–85%.

Sharma et al. developed a straightforward method to synthesize a series of steroidal spiro 1,2,4-triazolidin-3-ones **96** [52–54]. The spiro derivatives were selectively obtained as the unique isomers by reacting semicarbazones **95** with hydrogen peroxide at 0 °C. The reactions were conducted in chloroform, yielding all products in good yields (82–85%) (Scheme 28). The authors proposed a free radical mechanism facilitated by hydrogen peroxide, generating a primary radical at the terminal nitrogen atom -CO-HN^•^ which then adds to the carbon atom of the imino group. The reaction mechanism was substantiated by theoretical calculations. According to the mechanism, the heterocyclic ring closes by the attack of the bulky radical -CO-HN^•^ over the α steroidal side to circumvent the 1,3-diaxial interaction with the methyl group at C-10.

#### Spiro-1,3,4-oxadiazoline steroid

**Scheme 29:**
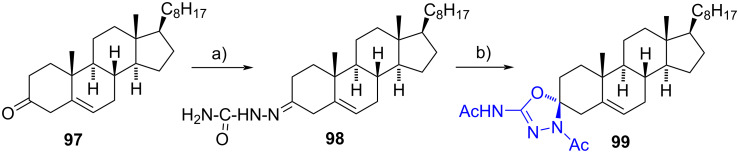
Steroidal spiro-1,3,4-oxadiazoline **99** obtained in two steps from cholest-5-en-3-one (**97**). a) NH_2_NHCONH_2_·HCl, NaOAc, EtOH/H_2_O, reflux, 73%; b) Ac_2_O, pyridine, CHCl_3_, 80 °C, 71%.

Shamsuzzaman et al. achieved the synthesis of 5’-acetamido-3’-acetyl-(3*R*)-spiro[cholest-5-en-3,2’-[1,3,4]-oxadiazoline] (**99**) through the acylation of semicarbazone **98** using freshly distilled acetic anhydride and pyridine at 80 °C [55]. Semicarbazone **98** was initially obtained via condensation of cholest-5-en-3-one (**97**) with semicarbazide hydrochloride in a refluxing ethanol–water–NaOAc mixture. Spiro heterocycle **99** was obtained in 52% overall yield as a single product (Scheme 29). The reaction mechanism was elucidated based on the hard and soft acid and base theory.

#### Spiro-1,3,4-thiadiazoline steroids

**Scheme 30:**
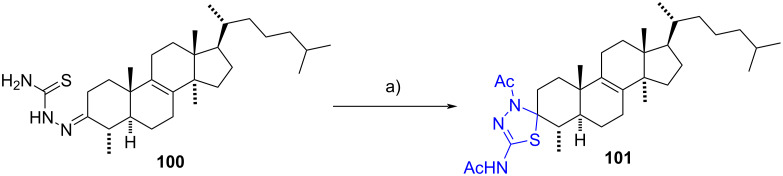
Synthesis of spiro-1,3,4-thiadiazoline **101** by cyclization and diacetylation of thiosemicarbazone **100**. a) Ac_2_O, pyridine, reflux, 64%.

In 2006, Mazoir et al. [56] reported the synthesis of 4α,14α-dimethyl-5α-cholest-8-en-3-one thiadiazoline **101** from thiosemicarbazone **100**. This last compound was treated with acetic anhydride in refluxing pyridine, resulting in the formation of the desired product in 64% yield (Scheme 30). No information was given about the stereochemistry at C-3.

More recently, steroidal mono- and bis(1,3,4-thiadiazolines) were described by Krstić’s group from the corresponding mono- and bis(thiosemicarbazones) [57]. Thiosemicarbazones were prepared by coupling estrane and androstane derivatives **102a**–**d** with thiosemicarbazide in refluxing ethanol and acetic acid.

**Scheme 31:**
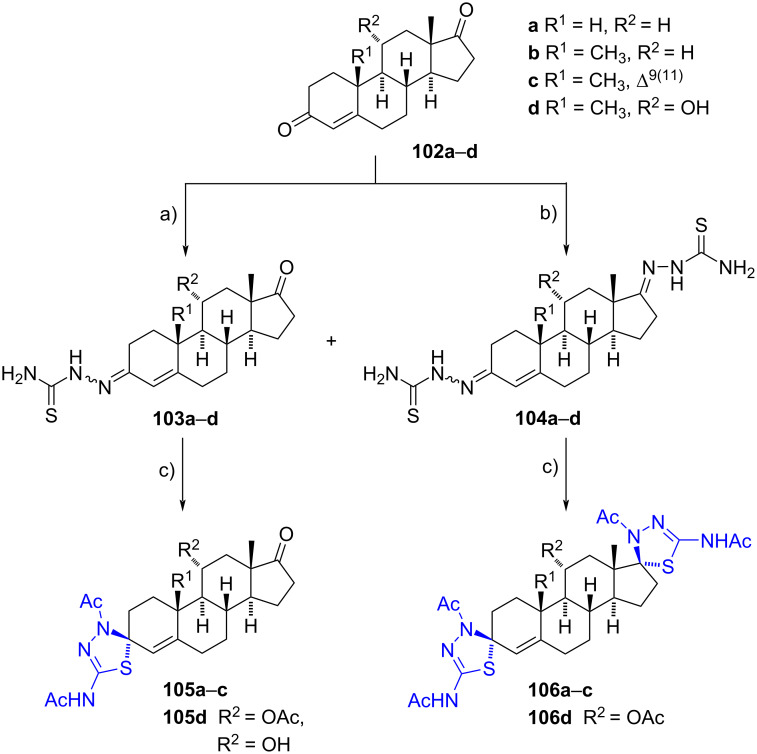
Mono- and bis(1,3,4-thiadiazolines) obtained from estrane and androstane derivatives. a) H_2_NCSNHNH_2_ (1.0 equiv), EtOH, reflux, 40–78%; b) H_2_NCSNHNH_2_ (2.0 equiv), EtOH, reflux, 48–56%; c) Ac_2_O, pyridine, CHCl_3_, 85 °C, **105a**–**c** (67–73%), **105d** [R^2^ = OAc (67%), R^2^ = OH (8%)], **106a**–**d** (56–70%).

Compounds **103** were primarily obtained when using 1.0 equiv of thiosemicarbazide (40–78% yields), although bis(thiosemicarbazones) **104** were also detected in small amounts (6–13% yield). Conversely, bis(thiosemicarbazones) **104** were predominantly obtained when 2.0 equivalents of thiosemicarbazide were used (48–56% yields), although 3-thiosemicarbazones **103** were still observed in minor quantities (6–11% yields). In both cases, thiosemicarbazones were obtained as a mixture of *E* and *Z* isomers, which were subsequently treated together with freshly distilled acetic anhydride and pyridine at 85 °C, yielding a single product (56–73% yield). The only exceptions were compounds **105d**, where hydroxy- and acetylated derivatives were isolated (Scheme 31).

Both thiosemicarbazones **103** and thiadiazolines **106** exhibited significant antiproliferative and pro-apoptotic activities against a panel of human cancer cell lines (K562, HeLa, MDA-MB-453, LS174, and A549). Among these, compound **106a** demonstrated the most promising properties. Based on these results, it was concluded that the presence of an α,β-unsaturated thiosemicarbazone moiety at C-3 or a spirothiadiazoline substituent at C-17 enhances the activity of the evaluated compounds.

#### Spiro-1,3,2-oxathiaphospholane steroids

**Scheme 32:**
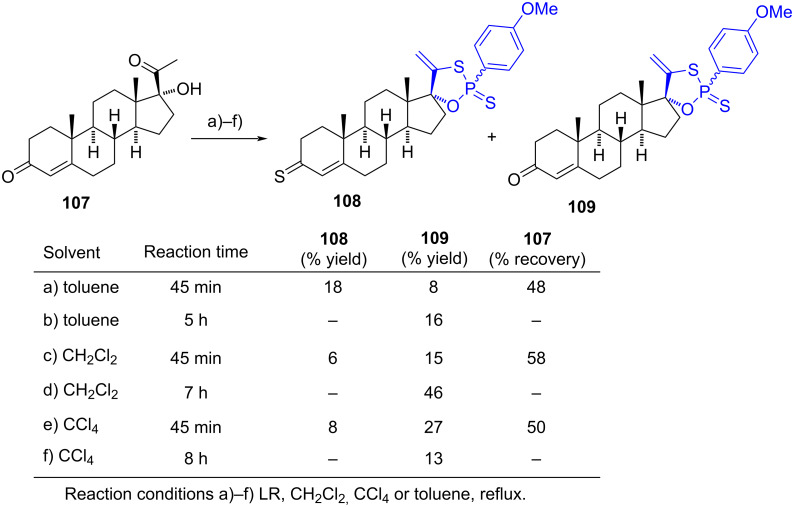
Different reaction conditions to synthesize spiro-1,3,2-oxathiaphospholanes **108** and **109**.

In 2012, Krstić et al. reported the synthesis of spiro-1,3,2-oxathiaphospholane steroids using Lawesson’s reagent (LR) as a thionating and phosphorylating agent in the reaction with 17α-hydroxyprogesterone (**107**) [58]. The reaction was conducted in toluene, dichloromethane, or carbon tetrachloride, and the resulting products varied depending on the reaction time. When the reaction was quenched at 45 min, two 17-spiro-1,3,2-oxathiaphospholane compounds **108** and **109** were isolated, besides a huge starting material; **108** bearing a 3-thioxo group (Scheme 32). Conversely, 3-oxoandrost-4-en-17-spiro-1,3,2-oxathiaphospholane (**109**) was obtained under all conditions. However, when the reaction time was increased to 5 h in toluene, no **108** was detected, but a higher yield of **109** was produced. The best yield (46%) for **109** was achieved after 7 hours of refluxing dichloromethane. Both compounds were obtained as a mixture of two diastereomers in a 7:3 ratio, with the phosphorus atom adopting different configurations. Only one isomer of **109** could be isolated.

The in vitro cytotoxicity of mixtures of isomers **108** and **109** was evaluated against three tumour cell lines: HeLa, MDA-MB-361, and MDA-MB-453, while antibacterial activity was tested against Gram-positive and Gram-negative bacteria. Unfortunately, all compounds exhibited very low activity in these assays. However, the oxathiaphospholanes were evaluated for their antifungal properties against *Candida albicans* and *Saccharomyces cerevisiae*, where the spiro compounds showed similar activity to that of nystatin (2.46–4.86 mM vs 0.89–2.70 mM for nystatin). Based on the comparable antifungal activity observed between **108** and **109**, the authors proposed that the oxathiaphospholane ring contributes to this bioactivity.

### Spiro steroids with six-membered heterocycles

#### Spirolactone steroids

**Scheme 33:**
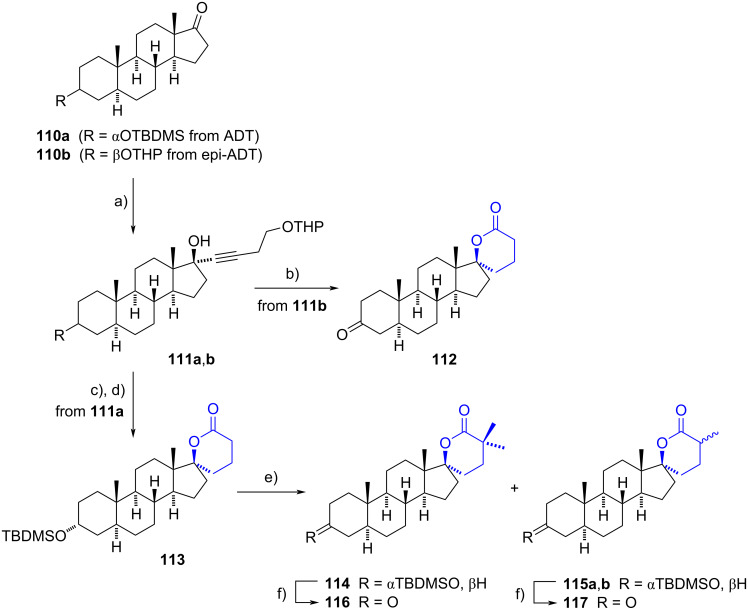
Spiro-δ-lactones derived from ADT and epi-ADT as inhibitors of 17β-HSDs. a) CH≡C(CH_2_)_2_OTHP, *n*-BuLi, THF, −78 °C, 68%; b) 1. H_2_, Pd/C, Pd/CaCO_3_, EtOAc, rt, 2. Jones reagent, acetone, 0 °C, 98%; c) 1. H_2_, Pd/C, Pd/CaCO_3_, EtOAc, rt, 2. *p*-TSA, MeOH, rt, 80%; d) Jones reagent, acetone, 0 °C, 95%; e) DIPA, *n*-BuLi, THF, CH_3_I, −78 °C to rt, 70% (**114**/**115a**/**115b** = 1:2:2); f) 1. TBAF, THF, reflux, 2. Jones reagent, acetone, 0 °C, **116** (92%), **117** (90%).

In 2013, Poirier’s group described a methodology to synthesize a series of spiro-δ-lactones as inhibitors of 17β-hydroxysteroid dehydrogenases (17β-HSD5 and 17β-HSD3) [42]. The synthesis began with the protection of androsterone (ADT) and epi-ADT with TBDMS or THP groups (**110a** and **110b**, respectively). These protected compounds were subjected to alkynylation using 4-THPO-1-butyne on the carbonyl group at C-17, yielding steroids **111**. Subsequent catalytic hydrogenation of the triple bonds, followed by deprotection of the alcohols from their THP ether groups and oxidation of the primary alcohols under Jones oxidation conditions, led to the corresponding carboxylic acids. These carboxylic acids underwent intramolecular cyclization with the 17β-hydroxy group to provide the spirolactones **112** and **113**. The ADT-lactone **113** was further methylated and oxidized to yield a mixture of three compounds, which were then oxidized at C-3 (Scheme 33). This synthetic pathway had previously been applied to TBDMS-protected estrone to achieve the 17-spiro-δ-lactone, which exhibited potent inhibition of 17β-HSD2 (IC_50_ = 6 nM) [18].

All the prepared compounds inhibited the activity of 17β-HSD5 in transfected HEK-293 cells (91–92% at 3 µM). The monomethylated lactone **117** showed better activity (73% at 0.3 µM) than the dimethylated compound **116** (54% at 0.3 µM). However, no inhibition was observed for 17β-HSD3. Interestingly, the compounds also demonstrated an antiproliferative effect on androgen-sensitive Shionogi cells, with weak binding to the androgen receptor detected. This suggests that the antiproliferative property may be attributed to antiandrogenic and cytotoxic activities. Moreover, the spiro derivatives did not show affinity to other steroid receptors, indicating a potentially favourable profile for prostate cancer therapy.

#### Spirolactam steroids

Steroidal spirolactams were obtained by Fousteris et al. in a five-step reaction sequence from 3β-benzyloxy-12β-hydroxydehydroepiandrosterone (**118**) [59]. The protected DHEA derivative was initially condensed with (*R*)-(+)-*tert*-butylsulfinamide, leading to the formation of the imine **119**. Subsequent reaction with allylmagnesium bromide yielded a mixture of 17-allyl 17-sulfonamido derivatives **120a**,**b** in a 2.5:1 diastereomeric ratio (17*S*:17*R*), which were isolated and treated separately. Acid treatment of the sulfinamides **120a**,**b** resulted in *tert*-butylsulfinyl cleavage, yielding the 17-allyl-17-amine hydrochlorides **121a**,**b** diastereomers. These hydrochlorides were then acrylated with acryloyl chloride.

**Scheme 34:**
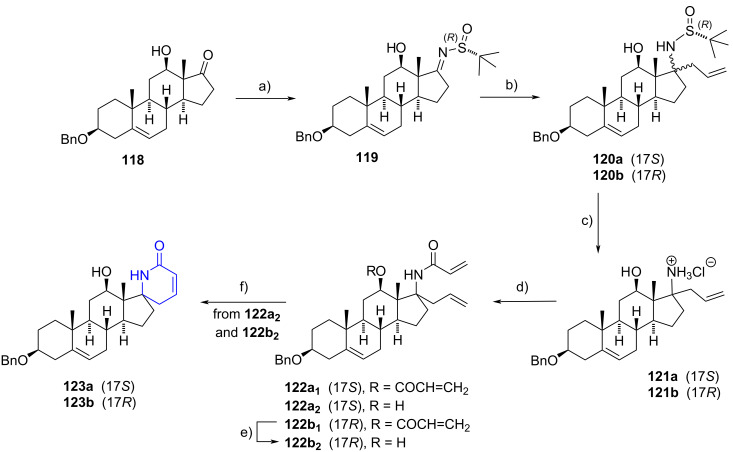
Spiro-δ-lactams **123a**,**b** obtained in a five-step reaction sequence. a) (*R*)-(+)-*tert*-butylsulfinamide, Ti(OEt)_4_, CH_2_Cl_2_, 50 °C, 75%; b) allylmagnesium bromide, CH_2_Cl_2_, –78 °C to rt, **120a** (50%), **120b** (19%); c) HCl, MeOH, rt, 87%; d) acryloyl chloride, Et_3_N, CH_2_Cl_2_/H_2_O, 0 °C to rt, **122a_1_** (3%), **122a_2_** (81%), **122b_1_** (59%), **122b_2_** (28%); e) NaOMe, MeOH, rt, 96%; f) G-II, CH_2_Cl_2_, reflux, **123a** (71%), **123b** (73%).

When starting from **121a**, the bis-acylated compound **122a_1_** was only detected in traces (3% yield), with the mono-acylated derivative **122a_2_** being the major product (81% yield). Conversely, using **121b** exclusively yielded the bis-acylated product **122b_1_** (59% yield), which was transformed into the mono-acylated compound **122b_2_** upon treatment with sodium methoxide. A final ring-closing metathesis (RCM) using a second-generation Grubbs catalyst (G-II) afforded the corresponding 17-spirolactams **123a**,**b** in 71–73% yield (Scheme 34). Notably, when (*S*)-(+)-*tert*-butylsulfinamide was used in the initial step of the reaction sequence, the allylation of the resulting imine proceeded diastereoselectively, yielding the 17-allyl-17-sulfinamido compound as the sole isomer.

#### Spiropiperidine steroids

**Scheme 35:**
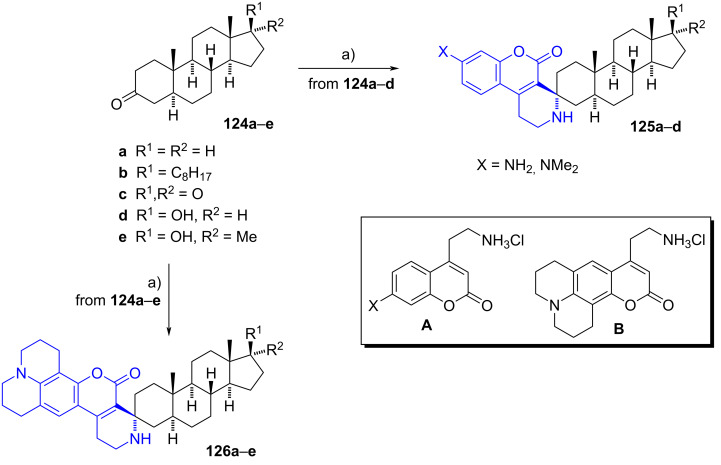
Steroid-coumarin conjugates as fluorescent DHT analogues to study 17-oxidoreductases for androgen metabolism in prostate cancer. a) Coumarin hydrochloride A or B, EtOH, conc. HCl, reflux, **125a**–**d** (21–85%), **126a**–**d** (19–86%).

Recently, Sviripa et al. synthesized novel spiro fused-coumarinpiperidines from 5α-androstan-3-one derivatives as part of their efforts to develop fluorescent inhibitors of 17-oxidoreductases for androgen metabolism in prostate cancer [60]. Starting from steroids **124a**–**e**, adducts **125a**–**d** and **126a**–**e** were easily obtained as single diastereomers via a Pictet–Spengler condensation with coumarins A and B (used as fluorophores) under acidic conditions (Scheme 35). Computational binding studies of compound **126c** in the ligand-binding pocket of enzyme 17β-HSD5 revealed that the adduct occupies the same position as the endogenous ligand 5α-androstane-3,17-dione.

#### Spirooxazinane steroids

**Scheme 36:**
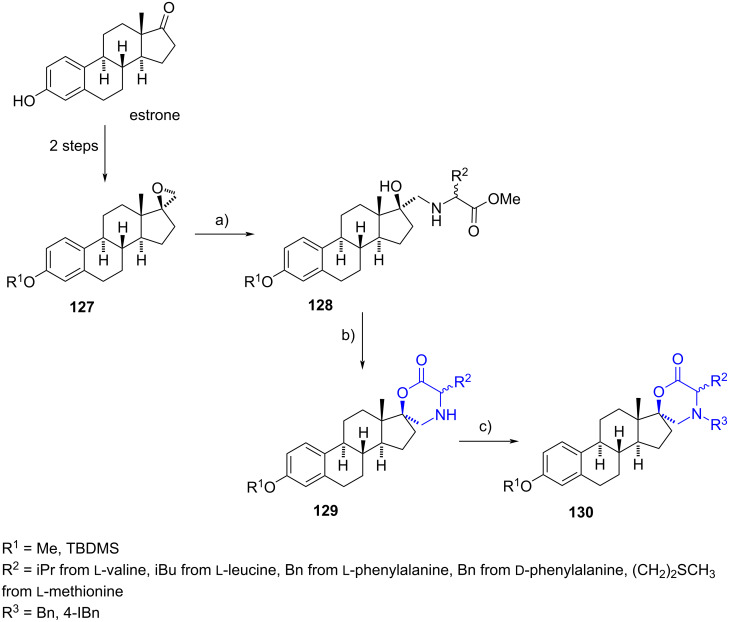
17-Spiro estradiolmorpholinones **130** bearing two types of molecular diversity. a) ʟ- or ᴅ-amino acid methyl ester, MeOH, Schlenk tube, 90–100 °C, 60–85%; b) NaH, THF, rt, 40–58%; c) DIPEA, BnBr or 4-IBnBr, CH_2_Cl_2_, Schlenk tube, 75 °C, 41–91%.

Spiromorpholinones are typically synthesized from 1,2-aminoalcohols, often derived from epoxides. In 2008, Poirier et al. reported several (17*S*)-spiro-(estradiol-17,2'-[1,4]oxazinan)-6'-one derivatives with two types of molecular diversity as part of their efforts to produce inhibitors of 17β-HSDs [61]. The first type of diversity was achieved by introducing different amino acids through the opening of the oxirane ring on compound **127**. After lactonization of compounds **128** promoted by the action of sodium hydride, alkylation of the morpholine nitrogen atom on **129** provided a diverse range of compounds (Scheme 36). In 2013, the same reaction sequence was reported by Poirier’s group, resulting in a spiromorpholinone at C-3 position derived from epi-ADT that exhibited activity as a 17β-HSD3 inhibitor [42].

**Scheme 37:**
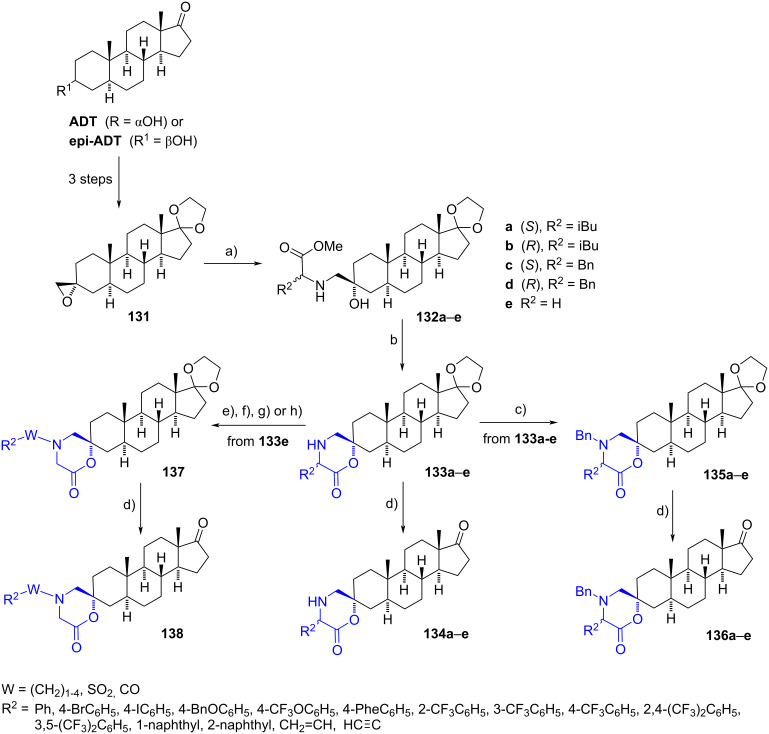
Steroidal spiromorpholinones as inhibitors of enzyme 17β-HSD3. a) Methyl ester of ʟ- or ᴅ-leucine, ʟ- or ᴅ-phenylalanine or glycine methyl esters, MeOH, 90 °C, 53–99%; b) NaOMe, THF, rt, 52–89%; c) DIPEA, BnBr, CH_2_Cl_2_, 75 °C, 49–70%; d) H_2_O/H_2_SO_4_, 1,4-dioxane, rt, **134a**–**e** (54–97%), **136a**–**e** (70–92%), **138** (28–94%); e) DIPEA, Br–W–R^2^, CH_2_Cl_2_, 65–75 °C, 41–95%; f) propargyl bromide, K_2_CO_3_, DMF, reflux, 65%; g) 4-(trifluoromethyl)benzenesulfonyl chloride, Et_3_N, CH_2_Cl_2_, rt, 78%; h) 4-(trifluoromethyl)benzoyl chloride, Et_3_N, CH_2_Cl_2_, rt, 86%.

The same research group reported a new family of 3-spiromorpholinones derived from ADT bearing hydrophobic substituents [43,62]. The synthetic key step involved a regioselective aminolysis of the oxirane **131** with different ʟ- or ᴅ-amino acids (53–99% yields) to obtain compounds **132a**–**e**. Spiro androstanemorpholinones **133a**–**e** were synthesized through the lactonization of the amino esters by treatment with sodium methoxide. Intramolecular transesterification was favored by a high volume of solvent and a low quantity of base, which helped to avoid undesired intermolecular side reactions. To increase the molecular diversity at the morpholine ring, tertiary amines were formed by nucleophilic substitution. A final removal of the cyclic ketal group in aq sulfuric acid provided spiromorpholinone derivatives **134a**–**e**, **136a**–**e**, and **138** (Scheme 37).

Spiromorpholinones were evaluated as inhibitors of 17β-HSD3 in a microsomal fraction of rat testes. Their androgenic effect on androgen-sensitive cells LAPC-4 was also evaluated. According to the biological results, compounds **134** and **136** with substituents in (*S*)-configuration were better inhibitors than the (*R*)-isomers. Furthermore, tertiary amines **136a**–**e** and **138** showed better 17β-HSD3 inhibition at 0.1 µM than the secondary ones **134a**–**e**, with some of them presenting better inhibition values than the reference compounds (RM-532-105 and D-5-2). However, only morpholinones **138** that bear sulfonamide and carboxamide groups did not exhibit androgenic activity.

**Scheme 38:**
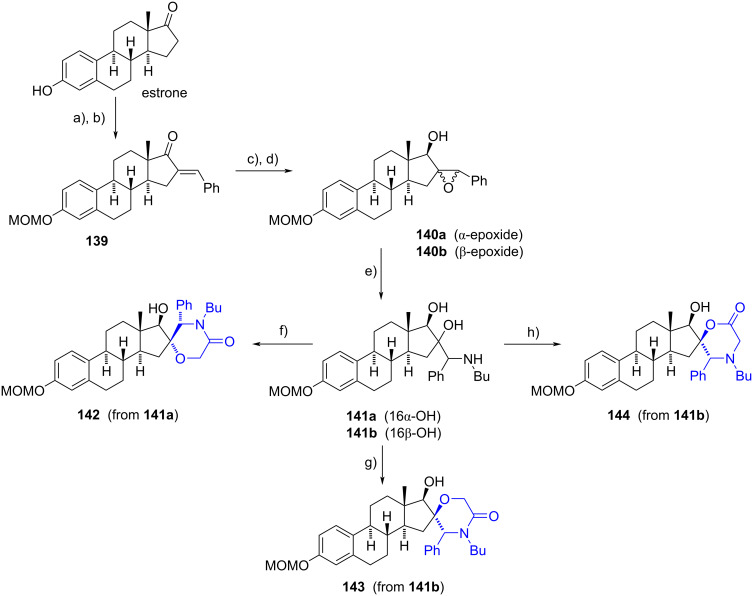
Steroidal spiro-morpholin-3-ones achieved by *N*-alkylation or *N*-acylation of amino diols **141**, followed by cyclization. a) MOMCl, DIPEA, CH_2_Cl_2_, rt; b) benzaldehyde, KOH aq, EtOH, 80 °C, 79%; c) NaBH_4_, MeOH/CH_2_Cl_2_, rt, 95%; d) *m*-CPBA, CH_2_Cl_2_, rt, **140a** (31%), **140b** (50%); e) BuNH_2_, EtOH, MW 170–180 °C, **141a** (65%), **141b** (99%); f) 1. ClCH_2_COCl, Et_3_N, CH_2_Cl_2_, rt, 2. KOH, EtOH, 60 °C, 40%; g) 1. ClCH_2_COCl, Et_3_N, THF, rt, 2. NaOMe, NaI, 80 °C, 21%; h) 1. BrCH_2_COOMe, Et_3_N, THF, rt, 2. K_2_CO_3_, MW 160 °C, 24%.

Poirier’s group reported two methodologies to prepare spiromorpholin-3-ones from a β-amino alcohol functionality [39]. Spiro compounds were synthesized starting from estrone, in which the phenol group was protected as a methoxymethyl ether. Then, the protected compound was subjected to an aldol condensation with benzaldehyde using potassium hydroxide in refluxing ethanol. The resulting enone **139** was reduced with sodium borohydride in methanol to give diastereoselectively the 17β-allylic alcohol. A successive treatment with *m*-CPBA in dichloromethane provided a mixture of epoxides **140a**,**b** (α/β 35:65), which were successfully separated. The following aminolysis of the isolated compounds was achieved by microwave heating using 3 equivalents of butylamine in ethanol at 180 °C. Finally, formation of morpholinones **142** and **143** was accomplished by *N*-acylation of the corresponding amino diol **141** with chloroacetyl chloride, followed by cyclization induced by formation of the alcoholate. On the other hand, amino diol **141b** was *N*-alkylated with methyl bromoacetate and then microwaved in the presence of potassium carbonate to obtain morpholinone **144** (Scheme 38).

**Scheme 39:**
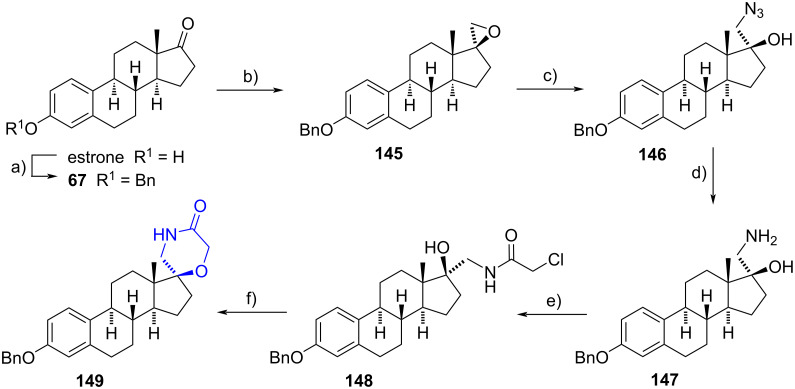
Straightforward method to synthesize a spiromorpholinone derivative from estrone. a) BnBr, K_2_CO_3_, CH_3_CN, reflux; b) SMe_3_I, *t*-BuOK, DMF; c) NaN_3_, H_3_BO_3_, DMF, reflux, 87% from estrone; d) LiAlH_4_, THF, reflux, 64%; e) ClCH_2_COCl, Et_3_N, MeOH–CH_2_Cl_2_, 51%; f) *t*-BuOK, DMF, 43%.

Romero-Hernández and Merino-Montiel recently reported a straightforward method to obtain the spiromorpholinone cycle on estrone. Initially, estrone was benzylated at C-3 and subjected to a Corey–Chaykovsky reaction using trimethylsulfonium iodide and potassium *tert*-butoxide, to produce diastereoselectively the epoxide **145**. The epoxide was then opened by treatment with sodium azide and boric acid, yielding the azide derivative **146** in 87% from estrone. A subsequent reduction of the azide with LiAlH_4_ provided the aminoalcohol derivative **147** in 64% yield. Then, a chloroacetamido moiety was formed at the amino function in 51% yield by using chloroacetyl chloride in the presence of triethylamine. The cyclization occurred when potassium *tert*-butoxide was employed to produce the substitution of the chloride atom by the hydroxy group at C-17. Compound **149** was purified in 43% yield (Scheme 39). The spiro compound showed potent antiproliferative activity when tested against a panel of different tumorous cells (GI_50_ = 2.0–11 μM). Docking simulations revealed that spiromorpholinone **149** could act as an aromatase inhibitor [63].

#### Spirotriazine steroids

**Scheme 40:**
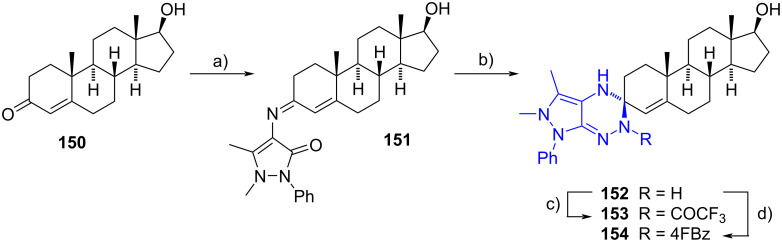
Pyrazolo[4,3-*e*][1,2,4]-triazine derivatives **152**–**154**. a) 4-Aminoantipyrine, EtOH/DMF, reflux, 82%; b) hydrazine, EtOH, piperidine, reflux, 75%; c) TFAA, THF, reflux, 62%; d) 4-fluorobenzoyl chloride, DMF, reflux, 55%.

Bakhotmah and Abdel-Rahman developed steroidal spiropyrazolotriazine derivatives **152**–**154** from 17α-hydroxyandrost-4-en-3-one (**150**) in a two-step sequence [64]. An initial condensation between 4-aminoantipyrine and the carbonyl group of **150** led to the imine **151**. Then, treatment of the latter with hydrazine provoked an addition to the imine carbon, followed by a dehydrative cyclization. To induce a more bioactive compound, fluorine atoms were introduced by acetylation and benzoylation of -NH at **152**. Spiro compounds **152**–**154** were easily afforded in 34–62% overall yields (Scheme 40). The compounds showed excellent coenzymatic effects towards cellobiase activity produced by *Chaetomium thermophilum* and *Thermomyces lanuginosus* fungi. The activity was related to the presence of fluorine atoms, and the substitution of -NH with trifluoroacetyl group achieved the most active compound.

#### Spirothiadiazine steroids

**Scheme 41:**
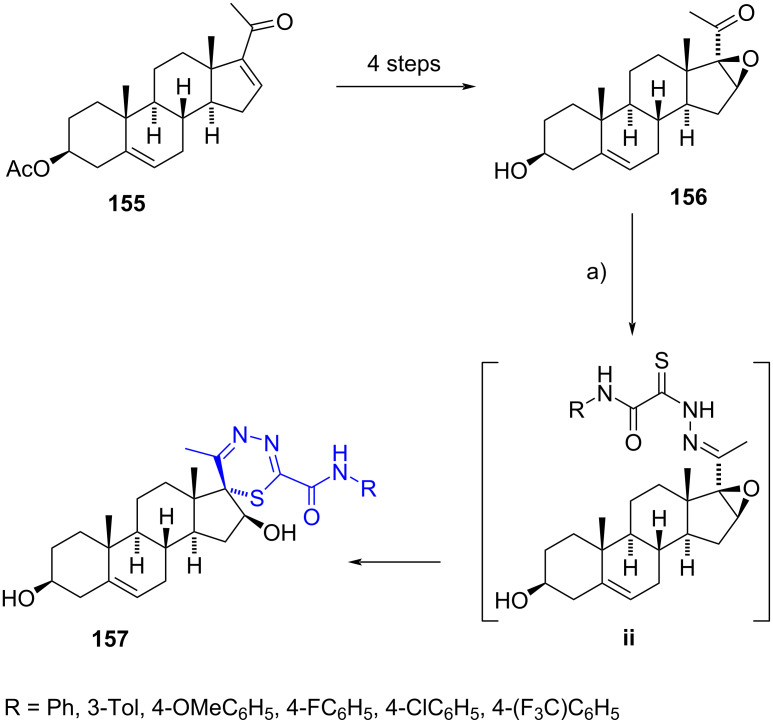
One-pot procedure to synthesize spiro-1,3,4-thiadiazine derivatives. a) NH_2_NHCSCONHR, H_2_SO_4_, dioxane, 60 °C, 51–63%.

Novel steroidal spiro-1,3,4-thiadiazines have been recently described through a one-pot reaction on 16β,17β-epoxypregnenolone (**156**), synthesized via a four-step process from 16-dehydropregnenolone acetate (**155**) [65]. Initially, the nucleophilic ring opening of the oxirane with substituted oxamic acid thiohydrazides led to a non-isolated intermediate hydrazone **ii**. Since both NH and SH are nucleophilic and can lead to the formation of five- or six-membered rings, the authors proposed that the thione group of **ii** was tautomerized to an iminothiol, from which the sulfur atom attacked the most electrophilic site of the epoxide (which was activated by protonation), producing the spiro-1,3,4-thiadiazine derivatives **157** in a stereoselective fashion (Scheme 41). The title compounds were screened for antiproliferative activity against the prostate cancer cell line 22Rv1, which unfortunately showed low values.

#### Spirotrioxane steroids

**Scheme 42:**
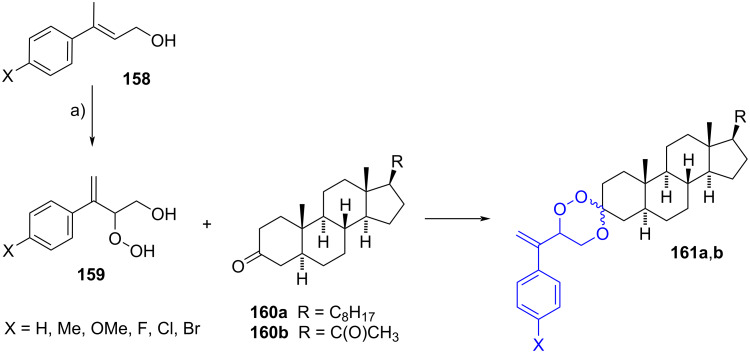
1,2,4-Trioxanes with antimalarial activity. a) 1. O_2_, methylene blue, CH_3_CN, 500 W tungsten halogen lamp, –10 to 0 °C; 2. steroid, HCl, rt, 23–87%.

The synthesis of spiro-1,2,4-trioxanes was described by Singh et al. through the photooxygenation of allylic alcohols **158** to produce the β-hydroxyhydroperoxides **159** [66]. The latter were reacted in situ with 3-ketosteroids **160a**,**b** in acidic medium to achieve trioxanes **161a**,**b** in moderate to good yields, but as an inseparable mixture of diastereomers (Scheme 42). The bioassays against multi-drug resistant parasite *Plasmodium yoelii* in Swiss mice revealed that pregnane-based trioxanes **161b** were the most active compounds, showing 100% suppression of parasitemia on day 4 at 48 mg/kg × 4 days, a similar activity to that of β-arteether used as positive control.

#### Bis-spirotetraoxane steroids

**Scheme 43:**
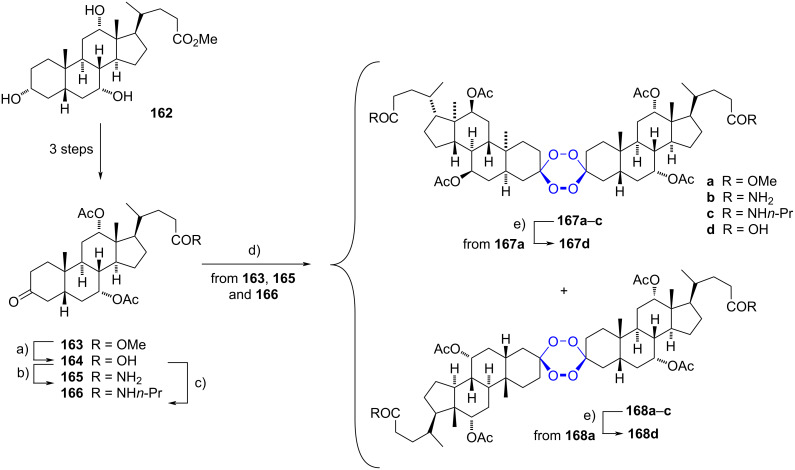
Tetraoxanes **167** and **168** synthesized from ketones **163**, **165** and **166**. a) NaOH, iPrOH/H_2_O, 80 °C, 93%; b) 1. benzene, SOCl_2_, reflux, 2. NH_4_Cl, Et_3_N, CH_2_Cl_2_, 0 °C to rt, 61%; c) 1. benzene, SOCl_2_, reflux, 2. *n*-PrNH_2_, CH_2_Cl_2_, 68%; d) toluene, EtOH/H_2_O/H_2_SO_4_, H_2_O_2_, 0 °C (for **163**) or TMSOTf/TMS_2_O_2_ (for **165** and **166**), **167a**–**c** (14–26%), **168a**–**c** (11–28%); e) NaOH, CH_2_Cl_2_/MeOH, rt, **167d** (79%), **168d** (72%).

Symmetrical bis-spiro tetraoxanes have been constructed by Milhous and Šolaja using methyl cholate (**162**) as starting material [67]. To produce the spiro compounds, the required ketone at C-3 was obtained in three reaction steps from **162** in 67% yield. A selective hydrolysis of the methyl ester under basic conditions was carried out, followed by the transformation of the carboxyl group to the amides **165** and **166** by treatment with thionyl chloride and the corresponding amine. Tetraoxanes were obtained from ketones **163**, **165**, and **166** by a peroxyacylation reaction using an acidic hydrogen peroxide solution or a mixture of bis(trimethylsilyl)peroxide and trimethylsilyl trifluoromethanesulfonate. In any case, the spiro products were afforded as a mixture of diastereomers. Finally, tetraoxanes **167d** and **168d** were synthesized by basic hydrolysis of the methyl esters **167a** and **168a** (Scheme 43).

Spiro tetraoxanes were tested for their antimalarial activity. From the biological results, it was observed that compounds with an amide moiety were more active compared to those possessing an ester or a carboxylic group. It was observed that tetraoxanes with a primary amide were more active towards *Plasmodium falciparum* chloroquine-resistant W2 clone, whereas *N*-propylamides showed better activity against the D6 clone. Among them, compound **167c** had the best activity (IC_50_ = 9.29 nM against the D6 clone), almost as good as artemisinin (IC_50_ = 8.6 nM) used as control. Tetraoxanes were also screened for their antiproliferative activity against a panel of cancer cells, in which the activity of some compounds was close to that of *cis*-platinum in some cell lines. It should be noted that *N*-propylamide derivatives showed low cytotoxicity on cancer cells. Additionally, **167c** did not present cytotoxic activity on normal human peripheral blood mononuclear cells (PBMC).

In 2003, a direct transformation of tetraoxanes to amides via carboxylic acid was also reported by the same research group [68]. Some amides were synthesized using methyl glycinate, di-*n*-propylamine, and piperidine with significant cytotoxicity to Fem-X and HeLa cells.

**Scheme 44:**
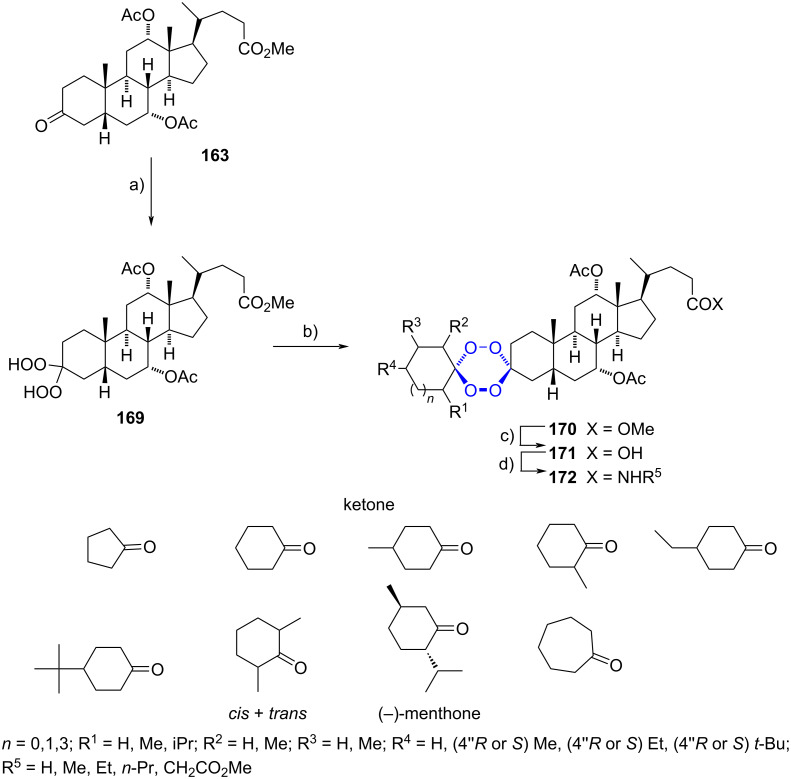
1,2,4,5-Tetraoxanes bearing a steroidal moiety and a cycloalkane. a) 30% H_2_O_2_/CH_2_Cl_2_/CH_3_CN, HCl, rt; 97%; b) ketone, H_2_SO_4_/CH_3_CN, CH_2_Cl_2_, 0 °C, 11–38%; c) NaOH, iPrOH/H_2_O, 90 °C, 72–89%; d) 1. Et_3_N, ClCO_2_Et, CH_2_Cl_2_, 0 °C; 2. R^5^NH_2_, rt, 64–96%.

In 2002, Šolaja et al. reported a straightforward method to synthesize non symmetrical steroidal tetraoxanes assembled by a steroid and a simple cycloalkane [69]. Initially, ketone **163** was converted to the steroidal *gem*-dihydroperoxide **169** with combined 30% hydrogen peroxide–hydrochloric acid. A subsequent reaction of the resulting compound with simple ketones in the presence of sulfuric acid produced steroidal 1,2,4,5-tetraoxanes **170** possessing an ester functionality. The unsubstituted ketones gave a single product as expected, while those with substituted precursors gave a diastereomeric mixture. A final transformation into the corresponding carboxylic acids **171** and amides **172** was performed in short reaction times (Scheme 44).

Steroidal tetraoxanes showed antimalarial activity. Twelve of them were more potent than artemisinin against *Plasmodium falsiparum* W2 clone resistant to chloroquine, with a 2.4-fold increase in activity for the most active compound compared to arteether, and a 6-fold increase compared to artelinic acid. Additionally, nine compounds exhibited better activity than chloroquine. Tetraoxanes were also evaluated on *Mycobacterium tuberculosis* with MIC values as low as 4.73 µM against H37Rv. In all biological tests, amides had better activities than esters and carboxylic acids. Moreover, mixed tetraoxanes showed improved results than bis-steroidal tetraoxanes previously synthesized by the same group. Some other derivatives have been reported with variations in the steroidal skeleton or in the cycloalkane residue, employing in all cases the methodology described before and tested as antimalarial, antiproliferative and antituberculosis agents [70–73].

#### Spirophosphorus steroids

**Scheme 45:**
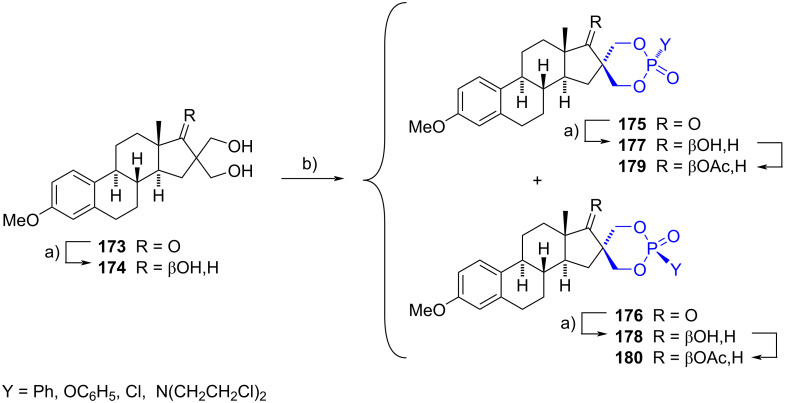
Spiro-1,3,2-dioxaphosphorinanes obtained from estrone derivatives. a) KBH_4_, MeOH, THF or CH_2_Cl_2_; b) YP(O)Cl_2_, Et_3_N, CH_2_Cl_2_, reflux, 12–91%.

In 2009, Frank et al. reported the preparation of diastereomeric pairs of 16-spiro-1,3,2-dioxaphosphorinanes [74]. The procedure started with the phosphorylation of 16,16-bis(hydroxymethyl)estrone 3-methyl ether (**173**) and its 17β-hydroxylated analog **174**, upon use of phosphorus(V) reagents and triethylamine. The transformations produced in almost all cases the diastereomeric mixtures that were successfully separated. The spiro compounds **175** and **176** were obtained from **173**, whereas **177** and **178** were afforded from **174**. The hydroxylated derivatives **177** and **178** could also be obtained by reduction with potassium borohydride of **175** and **176**, respectively. Finally, to facilitate their elucidation by NMR and to reduce the polarity of the molecules, the hydroxy functionality of **177** and **178** was acylated (Scheme 45).

Later, in 2012 the same research group applied the methodology to synthesize androstenedione derivatives which were tested as antiproliferative agents in three cancer cell lines (HeLa, MCF7, and A431). Unfortunately, only one spiro-1,3,2-dioxaphosphorinane exhibited values lower than 10 µM in two cell lines [75].

### Spiro steroids with seven-membered heterocycles

#### Spirolactone steroids

**Scheme 46:**
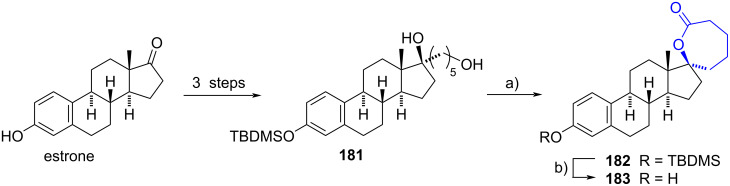
Synthesis of steroidal spiro-ε-lactone **183**. a) 1. Jones reagent, acetone, 0 °C to rt, 2. ClCOCOCl, DMF, CH_2_Cl_2_, rt, 3. pyridine, CH_2_Cl_2_, rt, 22%; b) Bu_4_NF, THF, 0 °C to rt, 40%.

In 2004 Poirier et al. reported the synthesis of a spiro-ε-lactone from the diol **181** obtained in turn from estrone after a 3-step sequence [18]. An initial oxidation reaction of the primary alcohol of **181**, under Jones conditions provided the carboxylic acid. Since no spontaneous lactonization was detected, the acid was reacted with oxalyl chloride to afford the corresponding acid chloride. After the addition of pyridine, cyclization was observed, giving the spiro-ε-lactone in 22% yield. The final compound **183** was obtained in 40% yield after cleavage of the silyl ether **182** (Scheme 46). Spiro-ε-lactone derivative was evaluated as an inhibitor of 17β-HSD2, presenting a low inhibitory value (IC_50_ = 150 nM).

#### Spirooxepine steroids

**Scheme 47:**
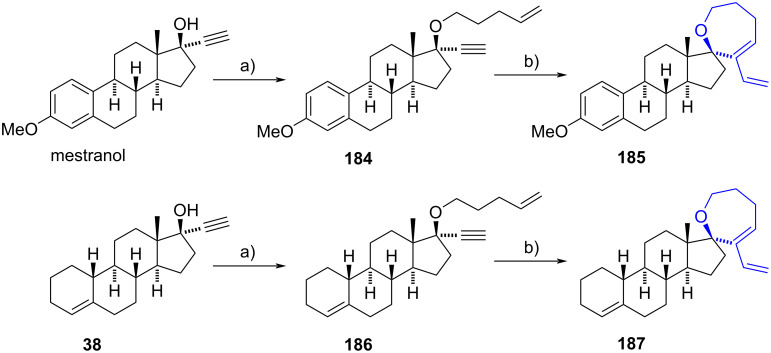
Synthesis of spiro-2,3,4,7-tetrahydrooxepines **185** and **187** derived from mestranol and lynestrenol (**38**). a) 4-Pentenyl bromide, DMF, NaH, 0 °C to rt, **184** (51%), **186** (49%); b) G-II, toluene, MW 170 °C, **185** (67%), **187** (80%).

In 2020, Nay’s group synthesized spirotetrahydrooxepines derived from mestranol and lynestrenol (**38**), following a procedure described before for the obtention of 2,5-dihydrofuran derivatives [25]. The 17α-ethynyl-17-hydroxysteroids were first alkylated at the hydroxy function with 4-pentenyl bromide obtaining the respective ethers **184** and **186** in moderate yields. A subsequent treatment of the afforded intermediates with the Grubb’s second-generation catalyst (G-II) at 170 °C conducted to a ring-closing enyne metathesis to give the spiro 2,3,4,7-tetrahydrooxepine moieties in 34 and 39% yields from mestranol and lynestrenol (**38**), respectively (Scheme 47).

## Conclusion

The recent advancements in the synthesis of steroidal spiro heterocycles highlight the versatility and biological potential of these compounds in medicinal chemistry. These spiro heterocycles feature various heterocyclic moieties attached to different positions of the steroidal backbone, with furans, pyrrolidines, oxazolines, thiazolidines, and oxazinanes being among the most frequently synthesized structures. While most reactions focus on constructing five- and six-membered rings, there are limited examples of four- and seven-membered heterocycles. Assigning the configuration of the new chiral centres in these compounds is crucial due to their significant bioactivity. Efficient methods to obtain single stereoisomers or to isolate diastereomers are necessary, considering that some spiro compounds are evaluated as mixtures. The biological activities of these molecules are diverse, ranging from antifungal, antimicrobial, and antimalarial activities to vasorelaxant effects and enzyme inhibition, notably against 17β-hydroxysteroid dehydrogenases implicated in hormone-dependent cancers. Antiproliferative activity against cancer cell lines is a common finding for many spiro heterocycles. Despite the promising therapeutic potential demonstrated by steroidal spiro heterocycles, research in this area is still relatively limited. The collected information underscores the need for further exploration and development of these compounds for various disease treatments. Overall, steroidal spiro heterocycles represent promising candidates in drug discovery and hold significant potential for future pharmacological applications.

## Data Availability

Data sharing is not applicable as no new data was generated or analyzed in this study.
